# Mechanical aspects of the semicircular ducts in the vestibular system

**DOI:** 10.1007/s00422-020-00842-w

**Published:** 2020-09-05

**Authors:** Mees Muller

**Affiliations:** 1 Experimental Zoology Group, Wageningen University, De Elst 1, 6708 WD Wageningen, The Netherlands; 2 Present Address: Physical Biology Institute Momchilovtsi, Ulica Bor 56, 4750 Momchilovtsi, Bulgaria

**Keywords:** Semicircular duct, Hair cells, Rotation receptor, Octavo-lateralis, Vestibular system

## Abstract

The semicircular ducts (SCDs) of the vestibular system play an instrumental role in equilibration and rotation perception of vertebrates. The present paper is a review of quantitative approaches and shows how SCDs function. It consists of three parts. First, the biophysical mechanisms of an SCD system composed of three mutually connected ducts, allowing endolymph to flow from one duct into another one, are analysed. The flow is quantified by solving the continuity equations in conjunction with the equations of motion of the SCD hydrodynamics. This leads to mathematical expressions that are suitable for further analytical and numerical analysis. Second, analytical solutions are derived through four simplifying steps while keeping the essentials of the coupled system intact. Some examples of flow distributions for different rotations are given. Third, the focus is on the transducer function of the SCDs. The complex structure of the mechano-electrical transduction apparatus inside the ampullae is described, and the consequences for sensitivity and frequency response are evaluated. Furthermore, both the contributions of the different terms of the equations of motion and the influence of Brownian motion are analysed. Finally, size limitations, allometry and evolutionary aspects are taken into account.

## Introduction and motivation

**Fig. 1:**
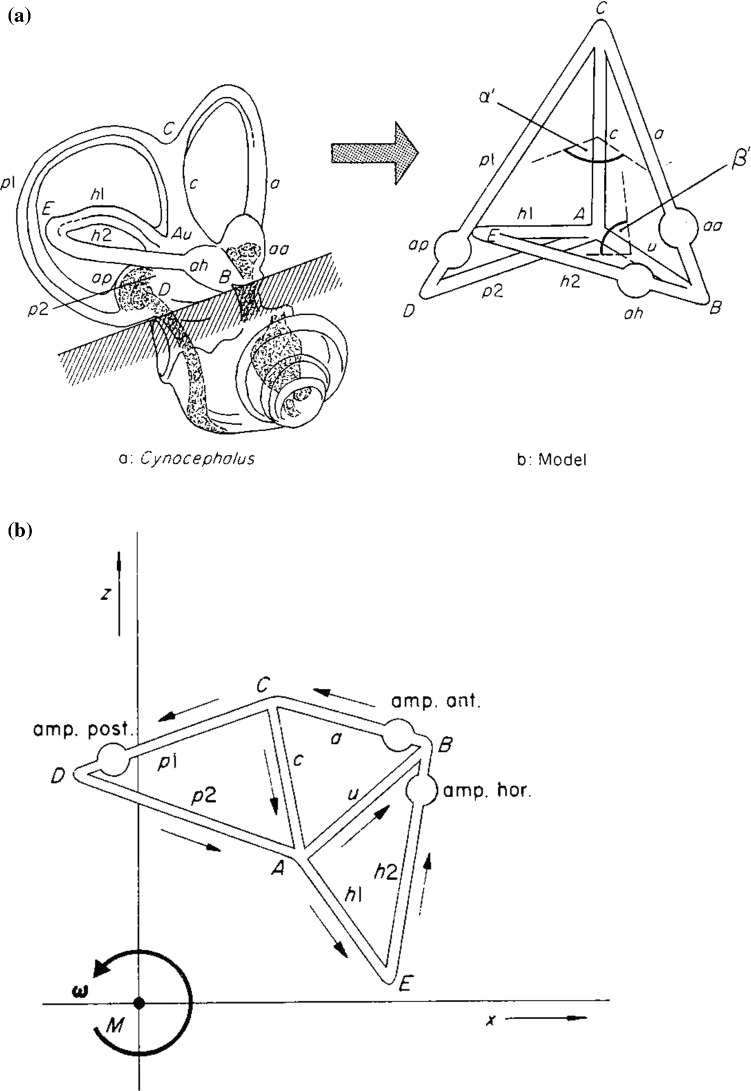
Construction of a three-dimensional semicircular duct (SCD) system. **A** The system in space: in a mammal and as a model. Abbreviations: a = anterior duct, aa = ampulla anterior, p1, p2 = posterior duct, ap = ampulla posterior, h1, h2 = horizontal duct, ah = ampulla horizontalis, c = crus commune, u = utriculus. **B** The system folded out in a flat plane. The arrows indicate the positive sign of the velocities. The system is rotated anticlockwise by an angular velocity **ω** as indicated in the origin M and suddenly stopped at *t *=* 0*

The *semicircular ducts* (SCDs) are the vertebrate sensors for three-dimensional rotation. The SCD organ is a part of the *labyrinth*, the latter being a cluster of several *hair bundle mechanoreceptors*. These receptors, which belong to the *octavo*-*lateralis* nerve system (8th brain nerves), can be grouped into two essentially different types. A first group are *statocyst*-like organs formed by the maculae of the utriculus and the sacculus (both small sacs that form the principal compartments of the labyrinth), using heavy particles—small stones, so to speak which are currently called otoliths or otoconia—that exert inertial forces on the hair bundles forming the top of the sensory cells. These organs sense gravitational and linear inertial accelerations. A second group are *hydrodynamic* receptors, in which fluid flow actuates on the hair bundles (Fritsch and Straka 2014). The SCDs belong to the latter group. The SCD organ consists of three mutually connected ducts (Fig. 1a) filled with *endolymph*, a fluid with physical properties close to those of water. At rotation, this endolymph pushes against the ampullary *mechano*-*electrical transduction system* of the hair cells of the SCDs, quite a complex organ that will be described in detail in Sect. 8 (Fig. 10). For more than a century, the SCDs have been considered as three separate fluid circuits positioned perpendicularly to each other.

As a student, I studied the morphology and histology of SCDs, mainly from fishes, noticing that they were always *mutually connected* allowing fluid to flow from one duct into another one, and positioned *obliquely* in the head and that, in many species, they were *not perpendicular* to each other. Later, I became impressed by the charm and simplicity of the current physical models but I also felt rather sceptical about many aspects of them. In this article, a discussion of the above features is given. I will also put forward some novel ideas that have not been published yet.

In the present review, it is not the aim to give an up-to-date overview of all the literature on this subject. I focus on my own modelling and refer to related articles for other details. To reduce its length, only essential mathematical expressions are presented. Details can be found in the papers listed in the References. Symbols are explained in the text and in the figure captions.

## A short history of semicircular duct modelling

The history of SCD modelling begins with the discovery by Flourens (1824, by ablation experiments in pigeons and followed by a description of their behaviour) that endolymph flow mediates the stimulation of the mechano-electrical transduction system and, in this way, gives information about head rotation. Flourens did not know that birds also possess a lumbar vestibular system, to control equilibrium of the body (discovered by Necker 2006), and that certainly had influence on the outcome of his experiments.

It lasted until about 1873 before the first physical models were published, simultaneously by Mach (1873), Crum Brown (1874), and Breuer (1873). These models did not include the elastic properties of the mechano-electrical transduction system. For elastic properties, several hypotheses have been proposed (cf. Sect. 8), starting with the rotation experiments of Steinhausen in 1933 on the pike (*Esox*). Steinhausen compared the deflection of the *cupula*, (an elastic valve-like structure of *mucopolysaccharides* in the ampulla) with a swinging door (“swinging-door” theory, see Sect. 8).

Based on Steinhausen’s data, Van Egmond et al. (1949) constructed a model of the SCD system of a single circular duct. The differential equation of this model contained an inertial term, a frictional term and an elastic term. For about 40 years, this model was the principal base for most studies. Oman et al. (1987) extended this model to a non-uniform cross section of the circuit. Muller and Verhagen (1988a, b) published the first three-dimensional model, composed of three mutually interconnected ducts. Rabbitt (1999) published an almost identical model, but also carried out some very useful experiments (Rabbitt et al. 1995), which will be discussed in Sect. 9.

## Geometry of the semicircular duct system and rotation

From now onwards, the dimensions of all equations will be denoted within square brackets. So, [m] means [metres].

Virtually all physical analyses of biological systems have to start with a geometrical abstraction. In the present case, a simplified rotating SCD system is placed in an earth-bound frame; see Fig. 1. A triangular semicircular duct ABC lying in a flat plane in space with Cartesian coordinates *x*, *y* and *z* of all three corner points *A*, *B* and *C* with respect to an origin *M* can be described by the following equations (Muller and Verhagen 1988b),

1a $$ \left( A \right)x + \left( B \right)y + \left( C \right)z + \left( D \right) = 0 $$

The coefficients (*A*), (*B*) and (*C*) are described by the following determinant equations,

1b $$ \left( A \right) = \left| \begin{aligned} y_{B} - y_{A} \quad z_{B} - z_{A} \hfill \\ y_{C} - y_{A} \quad z_{C} - z_{A} \hfill \\ \end{aligned} \right| $$

1c $$ \left( B \right) = \left| \begin{aligned} z_{B} - z_{A} \quad x_{B} - x_{A} \hfill \\ z_{C} - z_{A} \quad x_{C} - x_{A} \hfill \\ \end{aligned} \right| $$

1d $$ \left( C \right) = \left| \begin{aligned} x_{B} - x_{A} \quad y_{B} - y_{A} \hfill \\ x_{C} - x_{A} \quad y_{C} - y_{A} \hfill \\ \end{aligned} \right| $$

1e $$ \left( D \right) = - \left[ {x_{A} \left( A \right) + y_{A} \left( B \right) + z_{A} \left( C \right)} \right]\quad \left[ {\mathrm{m}} \right] $$

A system composed of three such triangular ducts in a flat plane is shown in Fig. 1b. The arrows in this figure indicate the positive directions of the fluid (endolymph) flow within the ducts. These triangular ducts can be mutually positioned under angles (Fig. 1a), thus forming a three-dimensional SCD system. This system is rotated in space around the origin M by a rotation vector **ω** [rad/s]. In Sects. 4 and 5, equations will be given to explain how, based on this geometrical construction of ducts, velocities and forces inside these ducts can be calculated.

## Initial velocities after rotation

In fact, the SCD system is dealing with three coordinate frames: (I) an earth-bound frame which is considered completely stationary, (II) the frame of the duct connected to the head and rotated around an origin *M* by a rotation vector **ω** and (III) the frame of the moving endolymph fluid inside the duct. As rotation starts, the fluid is set into motion, governed mainly by inertia and friction and, to a very small extent, by the elastic mechano-electrical transduction system. The acceleration of the fluid in frame (III) with respect to frame (I) has been described by Valentinuzzi 1967):

2 $$ {\mathbf{a}} = \frac{{{\mathrm{d}}^{2} {\mathbf{f}}}}{{{\mathrm{d}}t^{2} }} + {\varvec{\upomega}} \times \left( {{\varvec{\upomega}} \times {\mathbf{r}}} \right) + {\mathbf{r}} \times \frac{{{\mathrm{d}}{\varvec{\upomega}}}}{{{\mathrm{d}}t}} + {\mathbf{2}{\varvec{\upomega}} } \times {\mathbf{v}}_{{\mathbf{a}}} + {\mathbf{a}}_{{\mathbf{a}}} \quad [{\mathrm{m/s}}^{2} ] $$

where **a** is the acceleration of a point within the endolymph with respect to the earth-bound frame, **f** is the position vector of the moving origin in the earth-bound frame, the first term at the right-hand side is the acceleration of the moving frame with respect to the earth-bound frame, the second term is the centripetal acceleration, the third term the tangential acceleration, the fourth term the Coriolis acceleration and the fifth term the apparent acceleration of the endolymph point relative to the moving frame (II), and $\times$ indicates a vector or cross-product. The vector ${\mathbf{v}}_{a}$ denotes the linear velocity of an endolymph point with respect to the moving frame (II), while **a** is the corresponding acceleration. **ω** is the angular velocity of the rotating SCD system around an origin M in frame (I), and **r** [m] is the vector from the origin to a rotating point.

For a first analysis, the consideration of all terms of the above equation would make the analysis of endolymph movement unnecessarily complex. Both Muller and Verhagen (1988a, b) and Rabbitt et al. (1995) have circumvented the terms in Eq. (2) they did not want to consider by rather different approaches.

Rabbitt et al. stimulated the SCDs in the toadfish (*Opsanus*) mechanically with a glass indenter, thus causing endolymph flow. This has two advantages: (a) All endolymph accelerations (and thus forces) occur in frame (I). The two other coordinate frames coincide with frame (I). (b) It is possible to obtain frequency response curves.

Muller and Verhagen (1988a, b) initially rotated the SCD system for a relatively long time (i.e. much longer than the longest time constant *T*_1_, see below). At *t *= 0, they stopped the rotation instantaneously, generating a so-called pseudo-impulse stimulation. Yet, all frames of coordinates (I, II, III) coincide. The only acceleration of Eq. (2) that remains is the tangential acceleration ${\mathbf{r}} \times \frac{{{\mathrm{d}}{\varvec{\upomega}}}}{{{\mathrm{d}}t}}$, which is integrated over the practically infinitely short period [*t* = 0 − *ε*, *t* = 0 + *ε*] in which the SCD system is stopped, so as to obtain an initial endolymph velocity inside a semicircular duct section s:

3 $$ {\dot{\mathbf{x}}}_{\mathrm{s}} \left( 0 \right) = {\varvec{\upomega}} \times {\mathbf{r}}\quad \left[ {\mathrm{m/s}} \right] $$

**Fig. 2:**
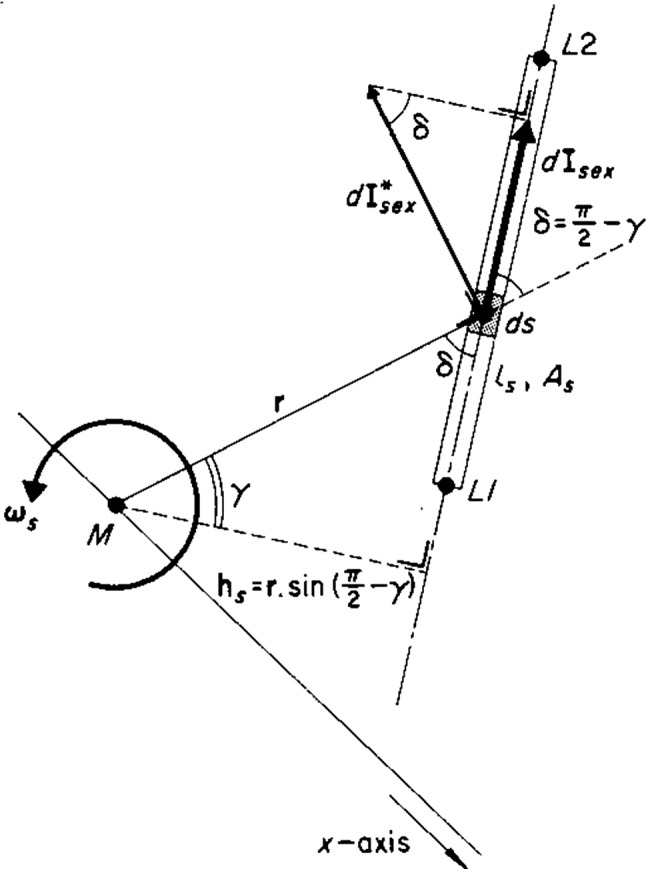
The external impulse **I**_**ex**_ in a duct part (s) stretching from L1 to L2, due to rotation by a vector **ω** in the origin M. This leads to Eq. (4) as explained in the main text

The subscript “s” stands for “section”, i.e. a particular part of the SCD system. This will be explained in more detail later in this section and is also indicated in Fig. 2.

Following Van Egmond et al. (1949), all single-duct theories have expressed mechanical quantities in rotational units. That is, angular displacement replaces linear displacement; analogously for angular velocity and angular acceleration, moment instead of force, moment of inertia instead of mass, etc. As the three-dimensional SCD system is composed of three mutually connected ducts, it contains two common parts, i.e. the crus commune c and the utriculus u (Fig. 1). When, for example, an anticlockwise rotation of endolymph fluid would be considered positive, a sign conflict for the endolymph movement in these common parts would occur. To understand this, let us consider Fig. 4A. When the fluid circuits ABC and ACD would be separate, the fluid in circuit ABC would flow from C to A. Analogously, in circuit ACD the fluid would flow from A to C. However, in the combined system, as drawn, the fluid direction in part CA (i.e. duct c) must be defined separately and independently of rotation. The same rules hold for duct u in Fig. 1. So, a linear instead of a rotational approach resolves the sign conflicts of Fig. 1B).

In this paper, endolymph movements are always considered with respect to the *moving* frame of coordinates of the duct walls. With laminar flow and, hence, without turbulence, the endolymph moves along the longitudinal axis of a duct, and perpendicularly to its cross-sectional area. This makes flow considerations effectively one-dimensional. The positive directions of endolymph velocities in different duct parts are given in Fig. 1B. Negative directions are opposite. Given the ducts, no other flow directions exist.

Secondary flow is not considered here but has been analysed in detail by Muller (1999).

Yet, it is useful to also define *normal vectors* **n**. With reference to Fig. 1, these vectors are unit vectors orthogonal to cross-sectional areas and their direction coincides with the velocity vectors described above. They have length 1 and are dimensionless. They can be applied so as to convert other vectors to scalars while keeping their sign intact. Details will become self-explanatory as we go along.

The rotation of the SCD system in Fig. 1 induces in all its 5 parts an amount of motion or, better, momentum. We have called these momentums the “*external impulses*”, abbreviated by **I**_**ex**_. Accordingly, the external impulse in a particular duct part is the momentum exclusively due to the motion of that duct part. In the equations below, a duct part, or section *s*, is indicated by a(n extra) subscript “*s*”.

The external impulse **I**_**ex**_ in a straight duct section *s*, i.e. **I**_**sex**_ is given by the following formula (Fig. 2)

4 $$ {\mathbf{I}}_{{{\mathbf{sex}}}} = m_{s} {\dot{\mathbf{x}}}_{s} \left( 0 \right) = \left( {\rho A_{s} l_{s} } \right)\left( {{\varvec{\upomega}}_{s} \times {\mathbf{h}}_{s} } \right)\left( {\mathrm{sign}} \right)_{\mathrm{s}} \quad \left[ {{\mathrm{kg}}\;{\mathrm{m/s}}} \right] $$

(Muller and Verhagen 1988b) where *m*_*s*_ is endolymph mass, ${\dot{\mathbf{x}}}_{s} \left( 0 \right)$ is the initial velocity of an endolymph point, ρ is endolymph density, *A*_*s*_ is the cross-sectional area of the section, *l*_*s*_ is the length of the section, **ω**_s_ is the component of the rotation vector in the section, **h**_s_ is the vector perpendicular from the origin of rotation M to the straight duct section (if the section would be circular as in the case of Eq. (23), **h**_s_ would be the radius vector **r**, as in Eq. (3)), and (sign) is the sign of the impulse that has to be determined in dependence upon the position of the rotating section to the origin M. This need not be explained here in detail as it can be found in Muller and Verhagen (1988b).

In fact, **I**_**ex**_ is a rather novel quantity, rarely used in physics. The *real momentum* (“*internal impulse*”) in a duct part is determined by the external impulses of *all* labyrinth parts. The momentum equations of the separate duct parts can be mutually connected by considering the pressures in the points of confluence of the ducts (i.e. *p*_C_ and *p*_A_, respectively) which leads to three momentum equations (cf. Eq. (5)).

An example of coupling of the external momentums I_*ex_ in ducts p and c (see the corresponding first row in Eq. (5); * stands for duct a, p, h, c or u) is

4a $$ n \bullet \left( {{\mathbf{I}}_{\mathrm{p}} - {\mathbf{I}}_{\mathrm{pex}} } \right)/A_{\mathrm{p}} = n \bullet \left( {{\mathbf{I}}_{\mathrm{c}} - {\mathbf{I}}_{\mathrm{cex}} } \right)/A_{\mathrm{c}} = \left( {p_{\mathrm{C}} - p_{\mathrm{A}} } \right)\Delta t\quad \left[ {{\mathrm{kg/(m}}\;{\mathrm{s)}}} \right] $$

where Δ*t* is the very short time interval in which the SCD system is stopped. **n** is the normal unit vector defined above and • denotes the dot product (scalar product). *A*_p_ and *A*_c_ are the cross-sectional areas of ducts p and c, respectively (Fig. 1).

Yet, it is necessary to consider the continuity equations of the system, i.e. the conservation of mass. There are three points of confluence of the system, so it is sufficient to consider two of them. This leads to two continuity equations thus describing the distribution of the flow at the point of confluence from one duct into the two other ducts. Together with the three impulse equations described above, they can be combined into the following 5 × 5 matrix equations for the determination of the five initial velocities

5 $$ \left[ {\begin{array}{*{20}l} 0 \hfill & {{{m_{\mathrm{p}} } \mathord{\left/ {\vphantom {{m_{\mathrm{p}} } {A_{\mathrm{p}} }}} \right. } {A_{\mathrm{p}} }}} \hfill & 0 \hfill & { - {{m_{\mathrm{c}} } \mathord{\left/ {\vphantom {{m_{\mathrm{c}} } {A_{\mathrm{c}} }}} \right. } {A_{\mathrm{c}} }}} \hfill & 0 \hfill \\ 0 \hfill & 0 \hfill & {{{m_{\mathrm{h}} } \mathord{\left/ {\vphantom {{m_{\mathrm{h}} } {A_{\mathrm{h}} }}} \right. } {A_{\mathrm{h}} }}} \hfill & 0 \hfill & { - {{m_{\mathrm{u}} } \mathord{\left/ {\vphantom {{m_{\mathrm{u}} } {A_{\mathrm{u}} }}} \right. } {A_{\mathrm{u}} }}} \hfill \\ {{{m_{\mathrm{a}} } \mathord{\left/ {\vphantom {{m_{\mathrm{a}} } {A_{\mathrm{a}} }}} \right. } {A_{\mathrm{a}} }}} \hfill & {{{m_{\mathrm{p}} } \mathord{\left/ {\vphantom {{m_{\mathrm{p}} } {A_{\mathrm{p}} }}} \right. } {A_{\mathrm{p}} }}} \hfill & {{{m_{\mathrm{h}} } \mathord{\left/ {\vphantom {{m_{\mathrm{h}} } {A_{\mathrm{h}} }}} \right. } {A_{\mathrm{h}} }}} \hfill & 0 \hfill & 0 \hfill \\ { - A_{\mathrm{a}} } \hfill & 0 \hfill & {A_{\mathrm{h}} } \hfill & 0 \hfill & {A_{\mathrm{u}} } \hfill \\ { - A_{\mathrm{a}} } \hfill & {A_{\mathrm{p}} } \hfill & 0 \hfill & {A_{\mathrm{c}} } \hfill & {A_{\mathrm{c}} } \hfill \\ \end{array} } \right]\left[ \begin{aligned} \dot{x}_{\mathrm{a}} \left( 0 \right) \hfill \\ \dot{x}_{\mathrm{p}} \left( 0 \right) \hfill \\ \dot{x}_{\mathrm{h}} \left( 0 \right) \hfill \\ \dot{x}_{\mathrm{c}} \left( 0 \right) \hfill \\ \dot{x}_{\mathrm{u}} \left( 0 \right) \hfill \\ \end{aligned} \right] = \left[ \begin{aligned} \left( {{{I_{\mathrm{pex}} } \mathord{\left/ {\vphantom {{I_{\mathrm{pex}} } {A_{\mathrm{p}} - {{I_{\mathrm{cex}} } \mathord{\left/ {\vphantom {{I_{\mathrm{cex}} } {A_{\mathrm{c}} }}} \right. } {A_{\mathrm{c}} }}}}} \right. } {A_{\mathrm{p}} - {{I_{\mathrm{cex}} } \mathord{\left/ {\vphantom {{I_{\mathrm{cex}} } {A_{\mathrm{c}} }}} \right. } {A_{\mathrm{c}} }}}}} \right) \hfill \\ \left( {{{I_{\mathrm{hex}} } \mathord{\left/ {\vphantom {{I_{\mathrm{hex}} } {A_{\mathrm{h}} - {{I_{\mathrm{uex}} } \mathord{\left/ {\vphantom {{I_{\mathrm{uex}} } {A_{\mathrm{u}} }}} \right. } {A_{\mathrm{u}} }}}}} \right. } {A_{\mathrm{h}} - {{I_{\mathrm{uex}} } \mathord{\left/ {\vphantom {{I_{\mathrm{uex}} } {A_{\mathrm{u}} }}} \right. } {A_{\mathrm{u}} }}}}} \right) \hfill \\ \left( {{{I_{\mathrm{aex}} } \mathord{\left/ {\vphantom {{I_{\mathrm{aex}} } {A_{\mathrm{a}} + {{I_{\mathrm{pex}} } \mathord{\left/ {\vphantom {{I_{\mathrm{pex}} } {A_{\mathrm{p}} + {{I_{\mathrm{hex}} } \mathord{\left/ {\vphantom {{I_{\mathrm{hex}} } {A_{\mathrm{h}} }}} \right. } {A_{\mathrm{h}} }}}}} \right. } {A_{\mathrm{p}} + {{I_{\mathrm{hex}} } \mathord{\left/ {\vphantom {{I_{\mathrm{hex}} } {A_{\mathrm{h}} }}} \right. } {A_{\mathrm{h}} }}}}}}} \right. } {A_{\mathrm{a}} + {{I_{\mathrm{pex}} } \mathord{\left/ {\vphantom {{I_{\mathrm{pex}} } {A_{\mathrm{p}} + {{I_{\mathrm{hex}} } \mathord{\left/ {\vphantom {{I_{\mathrm{hex}} } {A_{\mathrm{h}} }}} \right. } {A_{\mathrm{h}} }}}}} \right. } {A_{\mathrm{p}} + {{I_{\mathrm{hex}} } \mathord{\left/ {\vphantom {{I_{\mathrm{hex}} } {A_{\mathrm{h}} }}} \right. } {A_{\mathrm{h}} }}}}}}} \right) \hfill \\ 0 \hfill \\ 0 \hfill \\ \end{aligned} \right] $$

The former three lines are impulse equations [kg/(m s)], and the latter two represent the equations of continuity [m^3^/s]. It is explained below, under Eq. (6), that the initial velocities are the boundary conditions that are needed to activate the equations of motion.

## Equations of motion

Having stated above the geometry and the kinematics of the SCD system, we now turn to its dynamics. We still consider a SCD system that is initially turned for a long period of time, and instantaneously stopped at *t* = 0, as described earlier. The endolymph flow is then determined by endolymph inertia (first term of Eq. (6a)), the Poiseuille friction inside the fluid (second term) and the elastic force that is exerted on the fluid by the mechano-electrical transduction system (third term).

For a single duct circuit, the equation of motion (EoM) is expressed by the following second-order differential equation (a *force* equation),

6a $$ M\ddot{x} + F\dot{x} + Sx = 0\quad \left[ N \right] $$

where *M* is the endolymph mass, *F* is the friction coefficient and *S* is the elasticity coefficient of the mechano-electrical transducer system

6b $$ M = \rho A_{\mathrm{d}} l_{\mathrm{d}} $$

6c $$ F = 8\pi \eta l_{\mathrm{d}} \quad \left[ {\mathrm{kg}} \right]\quad {\mathrm{ and }}\quad \left[ {\mathrm{kg/s}} \right] $$

where $l_{\mathrm{d}}$ is duct length, $A_{\mathrm{d}}$ is the duct cross-sectional area and η is the dynamic viscosity of endolymph.

We can solve the homogenous Eq. (6a) by specifying two boundary conditions, e.g. the position $x\left( 0 \right)$ at *t* = 0 and the velocity $\dot{\varvec{x}}\left( 0 \right)$ at *t *= 0. Activation of the homogenous equation in this way frees us from defining unknown external forces [in the right-hand side of (6a)]. Its solution, i.e. the position *x* of an endolymph point, and its derivatives are sums of two exponentials (e-powers). Each exponential is characterized by a *time constant* that indicates the time needed to increase or decrease from its initial to its final value.

The two time constants of this damped mass–spring system can be derived directly from the EoM,

6d $$ T_{1}^{{}} = {F \mathord{\left/ {\vphantom {F S}} \right. } S} $$

6e $$ T_{2}^{{}} = {M \mathord{\left/ {\vphantom {M F}} \right. } F}\quad \left[ {\mathrm{s}} \right] $$

From Eq. (6) it can easily be derived that

7 $$ T_{2} = \frac{{\rho A_{\mathrm{d}} l_{\mathrm{d}} }}{{8\pi \eta l_{\mathrm{d}} }} = \frac{{r^{2} }}{8\nu } $$

where *r* is the radius of the duct and *ν* is the kinematic viscosity of the endolymph (i.e. the ratio of dynamic viscosity and density).

From *t *= 0 onwards, the endolymph starts to move rapidly (following *T*_2_) until a maximum excursion is reached (after about *t *= 5*T*_2_). The maximal endolymph excursion is found by differentiation of the increasing exponential of the endolymph displacement. One approximately obtains

8 $$ x_{ \mathrm{max} } \approx \dot{x}(0)T_{2} \quad \left[ {\mathrm{m}} \right] $$

After this maximal excursion, the endolymph returns slowly to its original position (following *T*_1_).

The solution of (6a) and the determination of the quantities (6b, 6c, 6d, 6e), (7) and (8) have been reported by Muller (1999), who is mainly using Laplace transforms. Graphs of the endolymph displacement can be found in the Muller (1999) paper. The solution of second-order differential equations with constant coefficients is treated in numerous elementary university textbooks.

For a human-like system, the following values hold (Muller 1999):

$$ T_{1} \approx 20\;\left[ {\mathrm{s}} \right],\quad T_{2} \approx \, 5 - 10\;\left[ {\mathrm{ms}} \right],\quad x_{\mathrm{max} } \approx 10\;\left[ {\upmu{\mathrm{m}}} \right],\quad \nu \approx 10^{ - 6} \;[{\mathrm{m}}^{2} / {\mathrm{s}}] $$

[The author has borrowed the value of *T*_1_ from a variety of older cupulometry references (listed in Muller 1999). A more precise value of *T*_1_ is about 5 [s] for the SCD system alone, which has been measured in the squirrel monkey (Fernandez and Goldberg 1971). Nevertheless, in the present discussion, only the order of magnitude of *T*_1_ is important]. For the SCD system, the damping ratio *ζ* is in the order of *ζ *= 30, i.e. a heavily overdamped system (explained in Sect. 8.3).

*To conclude, for a step*-*like stimulus of a single duct SCD system, as described above, the endolymph moves from its resting position to a maximal position, following a time course characterized by T*_2_*. This can be considered as the measuring phase of this system. Next, the endolymph restores very slowly to its resting position again, thus caused by the elastic force that presses to the fluid. This restoring phase that is characterized by T*_1_ *acts as a mechanical memory.*

Analogously to the momentum Eq. (4a), the equations of motion for the separate parts of the system can be combined while taking advantage of the pressure differences between the points of confluence. An example of the result is (Muller and Verhagen 1988b):

9 $$ {{\left( {M_{\mathrm{p}} \ddot{x}_{\mathrm{p}} + F_{\mathrm{p}} \dot{x}_{\mathrm{p}} + S_{\mathrm{p}} x_{\mathrm{p}} } \right)} \mathord{\left/ {\vphantom {{\left( {M_{\mathrm{p}} \ddot{x}_{\mathrm{p}} + F_{\mathrm{p}} \dot{x}_{\mathrm{p}} + S_{\mathrm{p}} x_{\mathrm{p}} } \right)} {A_{\mathrm{p}} }}} \right. } {A_{\mathrm{p}} }} = {{\left( {M_{\mathrm{c}} \ddot{x}_{\mathrm{c}} + F_{\mathrm{c}} \dot{x}_{\mathrm{c}} } \right)} \mathord{\left/ {\vphantom {{\left( {M_{\mathrm{c}} \ddot{x}_{\mathrm{c}} + F_{\mathrm{c}} \dot{x}_{\mathrm{c}} } \right)} {A_{\mathrm{c}} }}} \right. } {A_{\mathrm{c}} }} = \left( {p_{\mathrm{C}} - p_{\mathrm{A}} } \right)\quad \left[ {\mathrm{Pa}} \right] $$

At the far right of this equation, we see pressures. So, contrarily to Eq. (6a), Eq. (9) is a *pressure* equation. The far left expression is the EoM in duct p (cf. Figure 1 and Eq. (6a)), divided by the cross-sectional area *A*_p_ of duct p. The left-hand side of the equation thus specifies the pressure difference between points C and A in Fig. 1. The middle part of the above equation is the EoM in duct c divided by the cross-sectional area *A*_c_ of duct c. In the latter duct, no elastic part S exists.

Three second-order differential equations, similar to Eq. (9), emerge for the three semicircular ducts a, p and h and for the common parts c and u (cf. Fig. 1). Together with the continuity equations (see the two lower lines of formula (5)), the whole system can be combined (analogously to formula (5)), leading to a 3 × 3-matrix equation à la (9) for the endolymph excursions *x*(*t*) in the three ducts a, p and h, as explained by Muller and Verhagen (1988b: Eqs. (6.1.9)–(7.1.3)).

At this stage, we assume three equal elasticity constants *S* of the three cupular systems in the three ampullae (Fig. 1). This condition can be relaxed straightforwardly in the future. However, information about *S* is rather scarce and also extremely hard to obtain.

The solution of the system of differential equations as specified above yields three equations for the endolymph excursions in the three ducts a, p and h. These are each composed of 6 terms (Muller and Verhagen 1988b),

10a $$ x_{a} = \sum\limits_{i = 1}^{6} {a_{i} \exp \left( { - {t \mathord{\left/ {\vphantom {t {T_{i} }}} \right. } {T_{i} }}} \right)} $$

10b $$ x_{p} = \sum\limits_{i = 1}^{6} {b_{i} \exp \left( { - {t \mathord{\left/ {\vphantom {t {T_{i} }}} \right. } {T_{i} }}} \right)} $$

10c $$ x_{h} = \sum\limits_{i = 1}^{6} {c_{i} \exp \left( { - {t \mathord{\left/ {\vphantom {t {T_{i} }}} \right. } {T_{i} }}} \right)\quad \left[ {\mathrm{m}} \right]} $$

where *a*, *b* and *c* are constants. The exponentials thus contain three short time constants of type (6e) and three long time constants of type (6d). It should be noticed that the flow in each duct is influenced by all six time constants. This considerably differs from the case of three independent ducts where the flow in each individual duct is characterized by two time constants, a short and a long one.

**Fig. 3:**
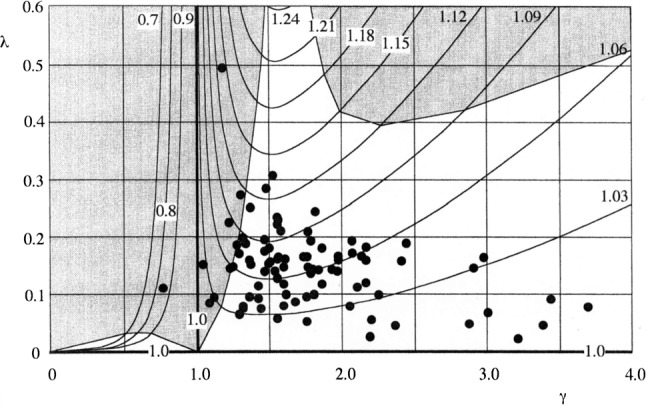
The function $f_{0} \left( {\gamma ,\lambda } \right)$. The contour lines indicate the values of this function for different *γ* and *λ*. Note that these values are close to 1. The dots are measurements for a variety of vertebrates after Muller and Verhagen (2002a)

The determination of the 6 constants a, the 6 constants b, the 6 constants c and the 6 time constants T in Eq. (10) requires the solution by means of an 18 × 18 matrix, which is not explained here but treated in detail by Muller and Verhagen (1988b: Eq. 7.1.3). Some important conclusions are:

*(1) Because the canal radii are approximately equal, the three short time constants are also approximately equal (see Sect.* *6**and Fig.* *3**).*

*(2) Two long time constants are approximately equal. The third one has about half the value of the two other ones as explained by Muller (*1990*).*

## Analytical solutions to the equations of motion

The above analysis of a three-duct SCD system always needs using a computer. It is, nevertheless, very helpful to attempt to simplify the system to obtain *analytical* solutions. The following simplifying steps can be performed.

### Simplifying step 1

A two-duct system is considered rather than a three-duct system. Although a two-duct system cannot give the same results as a three-duct one, a two-duct system keeps the essentials of a three-duct one intact. It represents a three-dimensional SCD system that has been rotated in space until t = 0. It also keeps the essentials of a hydrodynamically coupled system intact.

Because each duct contributes to the system with a second-order differential equation, this rigorously simplifies the system from a sixth-order one (three coupled ducts) to a fourth-order one (two coupled ducts). If necessary, the results of a two-duct SCD system can be checked against a numerical, computer-performed, calculation for a three-duct system.

### Simplifying step 2: only the fast process is considered

As stated already in Sect. 5, the fast process is the measuring phase of the system, and so the most important one. This simplification also eliminates the necessity to include the, rather unknown, elastic properties of the mechano-electrical transduction mechanisms. It is allowed as the fast and slow time constants differ a factor 1000 or more, as often happens in reality.

### Simplifying step 3: only the maximal endolymph excursions are considered

For a measuring instrument (think of a voltmeter), the final results are most relevant (i.e. the volts to be measured). The time course of the exponential functions is less or not even at all interesting (i.e. you usually wait till the volts to be measured are indicated).

### Simplifying step 4: the two ducts have equal lengths and radii

Measurements by Muller (1990, 1999) allow for this simplification. The length ratio of the ducts and the common part and the ratio of radii are defined to be:

11a $$ \lambda = \frac{{l_{\mathrm{c}} }}{{l_{\mathrm{d}} }} $$

11b $$ \gamma = \frac{{r_{\mathrm{c}} }}{{r_{\mathrm{d}} }} $$

Simplifying the system in this way leads to rather manageable expressions. It is even not necessary anymore to solve the 18 × 18 matrix for the endolymph excursions. To understand the latter statements, the reader should consult the detailed analysis in the original publications (Muller and Verhagen 2002a, b, c). The derivations require considerable algebra, effort and space.

Muller and Verhagen (2002a) have shown that six quantities are now important.

*(1) The fast time constants of the system:*

12 $$ \begin{aligned} T_{21} &= T_{22} \frac{{\left( {\left( {{{A_{\mathrm{c}}^{2} } \mathord{\left/ {\vphantom {{A_{\mathrm{c}}^{2} } {A^{2} }}} \right. } {A^{2} }}} \right){M \mathord{\left/ {\vphantom {M {F_{\mathrm{c}} + 2{{M_{\mathrm{c}} } \mathord{\left/ {\vphantom {{M_{\mathrm{c}} } {F_{\mathrm{c}} }}} \right. } {F_{\mathrm{c}} }}}}} \right. } {F_{\mathrm{c}} + 2{{M_{\mathrm{c}} } \mathord{\left/ {\vphantom {{M_{\mathrm{c}} } {F_{\mathrm{c}} }}} \right. } {F_{\mathrm{c}} }}}}} \right)}}{{\left( {\left( {{{A_{\mathrm{c}}^{2} } \mathord{\left/ {\vphantom {{A_{\mathrm{c}}^{2} } {A^{2} }}} \right. } {A^{2} }}} \right){M \mathord{\left/ {\vphantom {M {F_{\mathrm{c}} + 2{M \mathord{\left/ {\vphantom {M F}} \right. } F}}}} \right. } {F_{\mathrm{c}} + 2{M \mathord{\left/ {\vphantom {M F}} \right. } F}}}} \right)}}\\ &= T_{22} \frac{{\gamma^{2} \left( {\gamma^{2} + 2\lambda } \right)}}{{\left( {\gamma^{4} + 2\lambda } \right)}} = T_{22} f_{0} \left( {\gamma ,\lambda } \right)\quad \left[ {\mathrm{s}} \right] \end{aligned}$$

13 $$ T_{22} = \frac{M}{F} = \frac{{r^{2} }}{8\nu }\quad \left[ {\mathrm{s}} \right] $$

Note the similarity of Eqs. (7) and (13).

The function $f_{0}$ as defined by (12) is shown in Fig. 3. It can be observed that this function is close to 1 for all experimentally known values of *γ* and *λ*, as defined by Eq. (11). From Eq. (12), it then follows that the fast time constants are approximately equal. This can also be shown for a three-duct SCD system, as mentioned earlier.

*(2) The initial endolymph excursions in ducts a and p:*

14a $$  {\dot{\mathbf{x}}}_{{\mathbf{a}}} (0) = \frac{1}{M}\left[ {\frac{{\mathbf{I}_{{{\mathrm{aex}}}} (\gamma ^{2}  + \lambda )\mathbf{I}_{{{\mathrm{pex}}}} \lambda  + \mathbf{I}_{{{\mathrm{cex}}}} }}{{(\gamma ^{2}  + 2\lambda )}}} \right]\quad [{\mathrm{m}}]  $$

14b $$  {\dot{\mathbf{x}}}_{{\mathbf{p}}} (0) = \frac{1}{M}\left[ {\frac{{\mathbf{I}_{{{\mathrm{aex}}}} \lambda  + \mathbf{I}_{{{\mathrm{pex}}}} (\gamma ^{2}  + \lambda ) + \mathbf{I}_{{{\mathrm{cex}}}} }}{{(\gamma ^{2}  + 2\lambda )}}} \right]\quad [{\mathrm{m}}]  $$

and their ratio

15 $$ k_{\alpha } = \frac{{{\mathbf{n}} \bullet {\dot{\mathbf{x}}}_{\mathrm{p}} \left( 0 \right)}}{{{\mathbf{n}} \bullet {\dot{\mathbf{x}}}_{\mathrm{a}} \left( 0 \right)}} = \frac{{{\mathbf{n}} \bullet \left[ {{\mathbf{I}}_{\mathrm{aex}} \lambda + {\mathbf{I}}_{\mathrm{pex}} \left( {\gamma^{2} + \lambda } \right) - {\mathbf{I}}_{\mathrm{cex}} } \right]}}{{{\mathbf{n}} \bullet \left[ {{\mathbf{I}}_{\mathrm{aex}} \left( {\gamma^{2} + \lambda } \right) + {\mathbf{I}}_{\mathrm{pex}} \lambda + {\mathbf{I}}_{\mathrm{cex}} } \right]}} $$

*(3) The maximal endolymph excursions in ducts a and p:*

16a $$ \begin{aligned} x_{a\,\mathrm{max} } & = \dot{x}_{\mathrm{a}} \left( 0 \right)T_{22} f_{1} \left( {\gamma ,\lambda } \right) + \dot{x}_{\mathrm{p}} \left( 0 \right)T_{22} f_{2} \left( {\gamma ,\lambda } \right) \\ & = \dot{x}_{\mathrm{a}} \left( 0 \right)\left( {\frac{{T_{22} + T_{21} }}{2}} \right) + \dot{x}_{\mathrm{p}} \left( 0 \right)\left( {\frac{{T_{22} - T_{21} }}{2}} \right)\quad \left[ {\mathrm{m}} \right] \\ \end{aligned} $$

16b $$ \begin{aligned} x_{p\,\mathrm{max} } & = \dot{x}_{\mathrm{p}} \left( 0 \right)T_{22} f_{1} \left( {\gamma ,\lambda } \right) + \dot{x}_{\mathrm{a}} \left( 0 \right)T_{22} f_{2} \left( {\gamma ,\lambda } \right) \\ & = \dot{x}_{\mathrm{p}} \left( 0 \right)\left( {\frac{{T_{22} + T_{21} }}{2}} \right) + \dot{x}_{\mathrm{a}} \left( 0 \right)\left( {\frac{{T_{22} - T_{21} }}{2}} \right)\quad \left[ {\mathrm{m}} \right] \\ \end{aligned} $$

Or written in terms of external impulses **I**_**ex**_:

16c $$ x_{a\,\mathrm{max} } = \frac{{T_{22} }}{M}\left[ {\frac{{{\mathbf{n}} \bullet \left( {{\mathbf{I}}_{\mathrm{aex}} \left( {\gamma^{4} + \lambda } \right) + {\mathbf{I}}_{\mathrm{pex}} \lambda + {\mathbf{I}}_{\mathrm{cex}} \gamma^{2} } \right)}}{{\left( {\gamma^{4} + 2\lambda } \right)}}} \right]\quad \left[ {\mathrm{m}} \right] $$

16d $$ x_{p\,\mathrm{max} } = \frac{{T_{22} }}{M}\left[ {\frac{{{\mathbf{n}} \bullet \left( {{\mathbf{I}}_{\mathrm{aex}} \lambda + {\mathbf{I}}_{\mathrm{pex}} \left( {\gamma^{4} + \lambda } \right) - {\mathbf{I}}_{\mathrm{cex}} \gamma^{2} } \right)}}{{\left( {\gamma^{4} + 2\lambda } \right)}}} \right]\quad \left[ {\mathrm{m}} \right] $$

and their ratio

17 $$ k_{\alpha \mathrm{max} }^{{}} = \frac{{x_{p\,\mathrm{max} } }}{{x_{a\,\mathrm{max} } }}\,\, = \frac{{{\mathbf{n}} \bullet \left[ {{\mathbf{I}}_{\mathrm{aex}} \lambda + {\mathbf{I}}_{\mathrm{pex}} \left( {\gamma^{4} + \lambda } \right) - {\mathbf{I}}_{\mathrm{cex}} \gamma^{2} } \right]}}{{{\mathbf{n}} \bullet \left[ {{\mathbf{I}}_{\mathrm{aex}} \left( {\gamma^{4} + \lambda } \right) + {\mathbf{I}}_{\mathrm{pex}} \lambda + {\mathbf{I}}_{\mathrm{cex}} \gamma^{2} } \right]}} $$

**Fig. 4:**
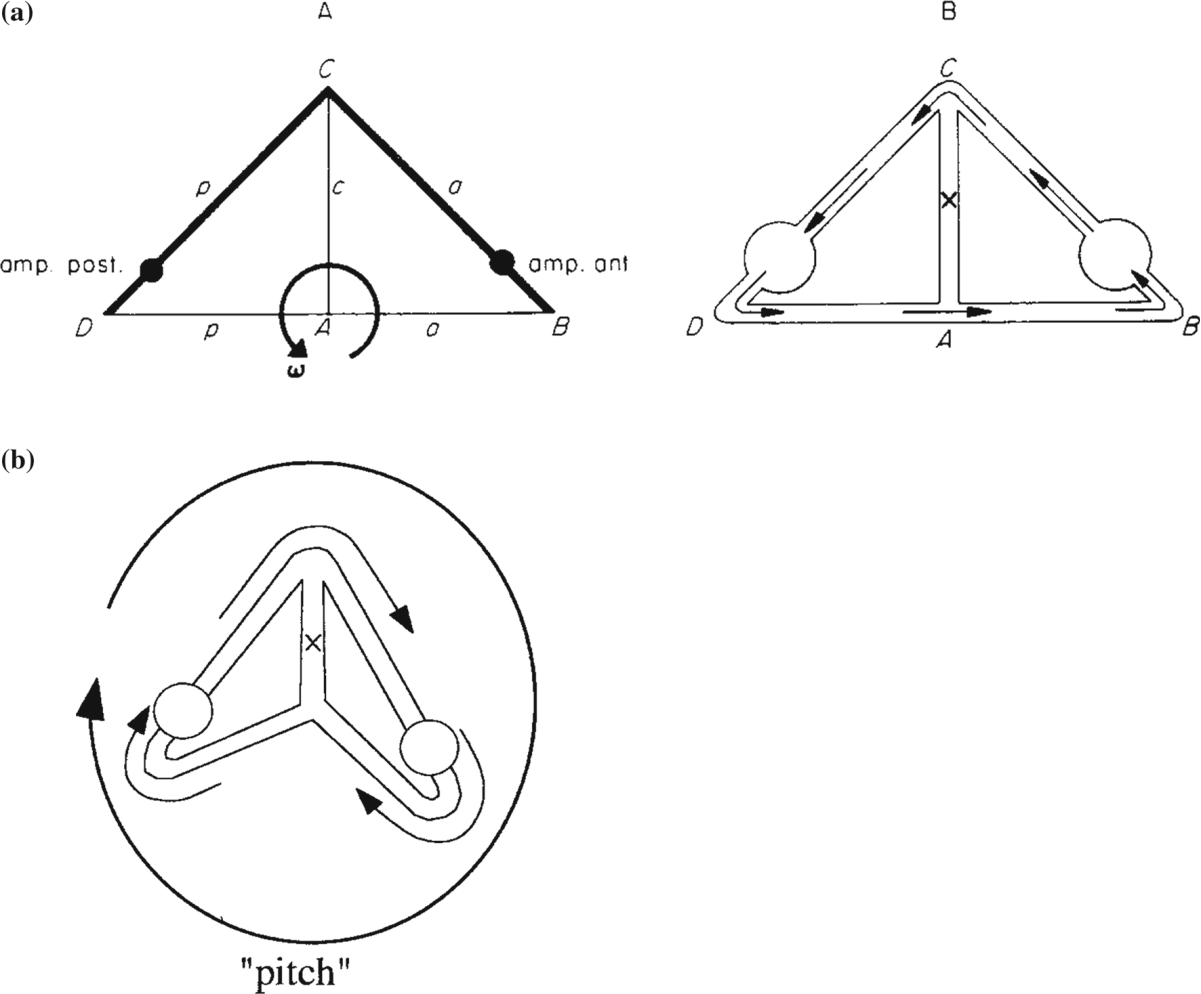
Serial coupling of ducts. Abbreviations are the same as in Fig. 1. In **AA**, the bold ducts indicate the parts wherein external impulses are generated. In **AB**, the resulting flow is shown. **B** gives a three-dimensional view of the flow. No flow exists in the common part

**Fig. 5:**
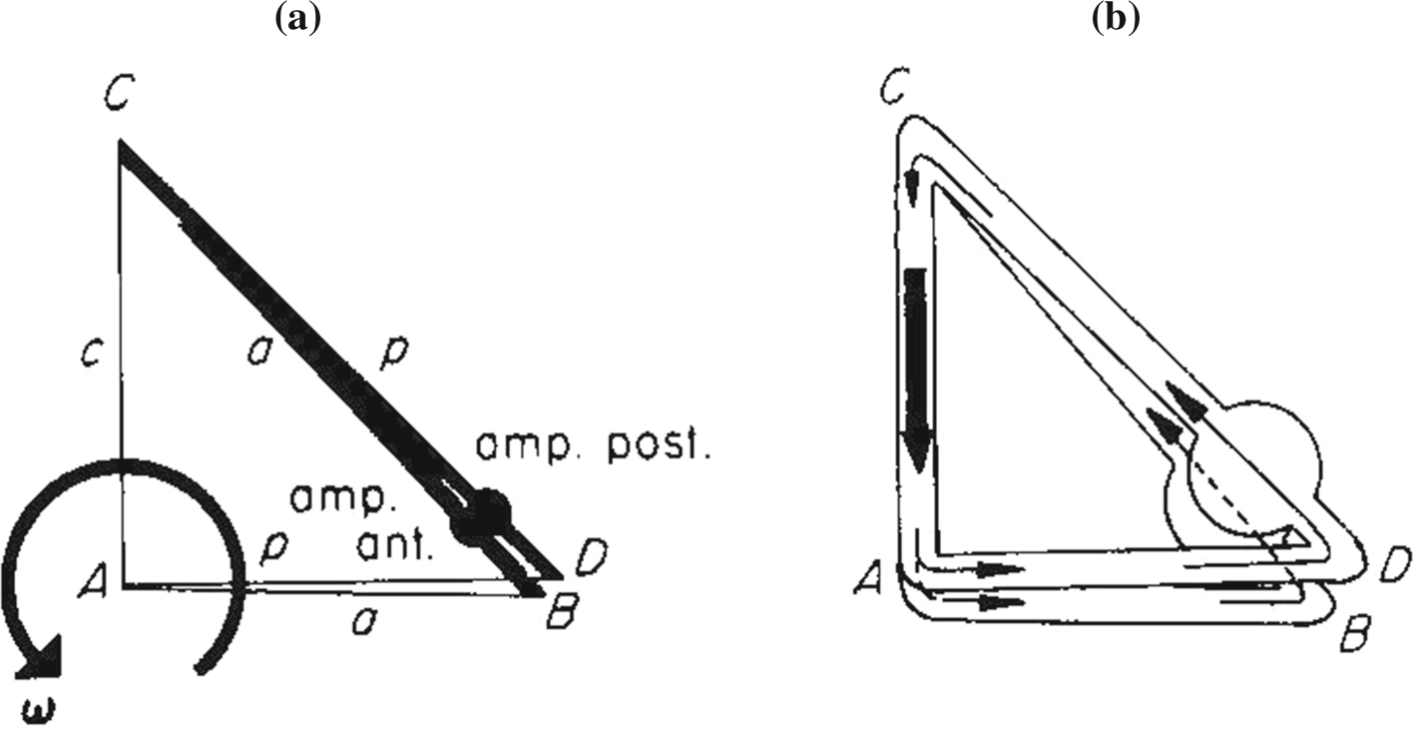
Parallel coupling of ducts. Abbreviations are the same as in Fig. 1. In **A**, the bold ducts indicate the parts wherein external impulses are generated. In **B**, the flow is shown. Now, in ducts a and p equal but opposite flows occur. In duct *c*, these flows are added

**Fig. 6:**
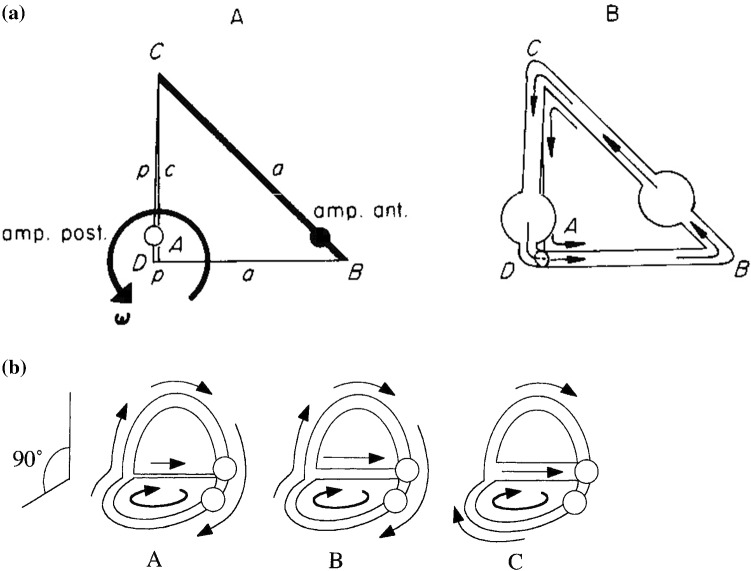
Coupling of perpendicular ducts. Abbreviations are the same as in Fig. 1. In **A**, the bold duct indicates the part wherein an external impulse is generated. In **B**, the flow is shown. The external impulse in duct a generates a Poiseuille flow in circuit ABC. This flow causes a pressure difference between points A and C. Consequently, also flow occurs in circuit ACD (duct p). In **B**, a three-dimensional picture of the flow is shown. Three situations are considered: (**A**) for a very narrow common part, (**B**) for a common part which has the same radius as the other parts, (**C**) for a very wide common part. In (**A**), almost the whole fluid flows through the ducts and hardly any flow is present in the common part. In (**C**), the flow in the vertical duct is much reduced

**Fig. 7:**
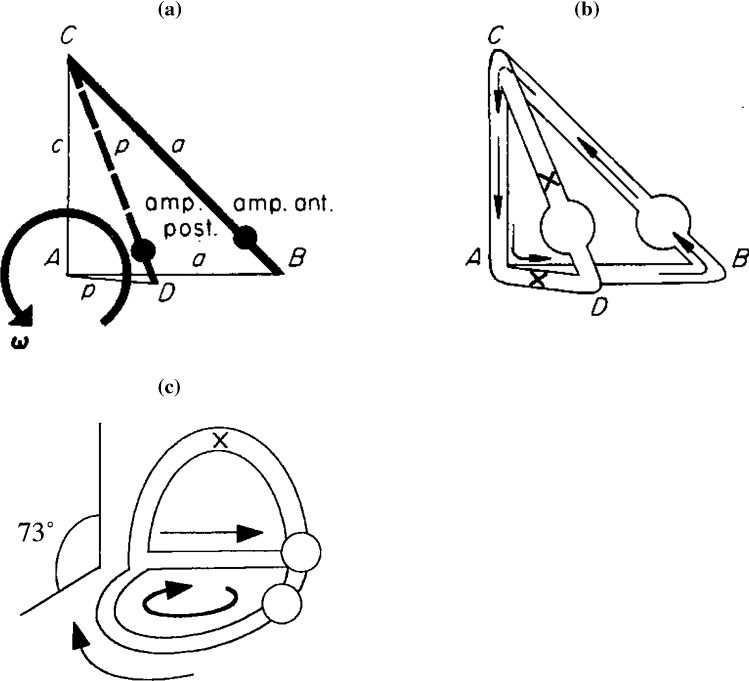
Coupling of ducts positioned obliquely. Abbreviations are the same as in Fig. 1. In **A**, the bold ducts indicate the parts wherein external impulses are generated. In **B**, the flow is shown. The external impulse in a is now counteracted by the external impulse in p. This impulse is unequal to the impulse in a and has opposite sign causing the flow in p to be zero. For the given geometry, the angle between the ducts is 73° (diagram **C**)

As has already been explained in Sect. 4, the normal vectors **n** in Eqs. (15)–(17) are dimensionless unit vectors that are used to create scalar products making all terms in the equations scalar. They are perpendicular to the cross-sectional areas of the ducts. In this way, the directions of the endolymph velocities are kept intact through the signs. Flow distributions will be treated in the next section and are shown in Figs. 4, 5, 6, 7.

*The above equations provide important features of the flow regime and the morphology of the SCD system. This is discussed in the next sections.*

## Some examples of flow distribution in the two-duct SCD system

Equations (15) and (17) can be used to evaluate the flow distribution in the SCD system. Although they obviously govern a two-duct system, they provide also useful ideas for a three-duct one. If one surmises that the flow in a three-duct system would considerably deviate from the flow in a two-duct one, one could check this by a computer simulation, as described in Sect. 5.

Several examples of interest are now going to be presented.

*(1) Serial coupling (Fig.* *4**)*

From Fig. 4a, it is known:

18a, b, c $$ M = M_{a} = M_{p} ,\quad {\mathbf{I}}_{\mathrm{pex}} = {\mathbf{I}}_{\mathrm{aex}} = {\mathbf{I}},\quad {\mathrm{I}}_{\mathrm{cex}} = 0 $$

From Eq. (15) it then follows

18d $$ k_{\alpha } = 1 $$

The flow regime is shown in Fig. 4b. In the common duct no flow exists. So, the anterior and posterior ducts are in series. The same rules for the anterior and the horizontal ducts when they are rotated in an analogous way. This fact will not be mentioned anymore.

*(2) Parallel coupling (Fig.* *5**)*

19a, b, c $$ M = M_{a} = M_{p} ,\quad {\mathbf{I}}_{\mathrm{pex}} = - {\mathbf{I}}_{\mathrm{aex}} ,\quad {\mathbf{I}}_{\mathrm{cex}} = 0 $$

From (15) we obtain

19d $$ k_{\alpha } = - 1 $$

Figure 5 exhibits the corresponding flow regime.

*(3) 90° coupling (Fig.* *6**)*

20a, b, c $$ M = M_{a} = M_{p} ,\quad {\mathbf{I}}_{\mathrm{pex}} = 0,\quad {\mathbf{I}}_{\mathrm{cex}} = 0 $$

Yet Eq. (15) gives

20d $$ k_{\alpha } = \frac{\lambda }{{\left( {\gamma^{2} + \lambda } \right)}} = f_{3} \left( {\gamma ,\lambda } \right) $$

For equal radii and the geometry shown in Fig. 4, it follows (Fig. 6A)

20e $$ \begin{aligned} {\dot{\mathbf{x}}}_{\mathrm{p}} \left( 0 \right) & = 0.29 \cdot {\dot{\mathbf{x}}}_{\mathrm{a}} \left( 0 \right) \\ {\dot{\mathbf{x}}}_{\mathrm{p}} \left( 0 \right) & \approx \left( {1 - k_{\alpha } } \right) \cdot {\dot{\mathbf{x}}}_{\mathrm{a}} \left( 0 \right) \approx 0.71 \cdot {\dot{\mathbf{x}}}_{\mathrm{a}} \left( 0 \right) \\ \end{aligned} $$

Equation (20e) nicely demonstrates that in duct p a flow is generated, if rotation takes place exclusively in the plane of duct a.

Figure 6B shows the flow for different values of *γ*. In Fig. 6BA, *γ* is very small. Thus, the flow generated in the horizontal duct (through rotation indicated by the inner arrow) continues (almost) entirely through the vertical duct. In Fig. 6BB, *γ* is larger and a coupled-flow regime occurs, as indicated in Fig. 6A. In Fig. 6BC, *γ* is very large and hardly any flow occurs in the vertical duct. The pressure difference between the points of confluence of the ducts is much reduced (shunted).

*(4) 73° coupling (Fig.* *7**)*

The question now arises under which condition no flow is generated in duct p when rotation takes place exclusively in the plane of duct a. We then find

21a, b, c $$ M = M_{\mathrm{a}} = M_{\mathrm{p}} $$

Substitution of these conditions in (15) reveals:

21d $$ \frac{{{\mathbf{n}} \bullet {\varvec{\upomega}}_{\mathrm{p}} }}{{{\mathbf{n}} \bullet {\varvec{\upomega}}_{\mathrm{a}} }} = - f_{3} \left( {\gamma ,\lambda } \right) \Rightarrow \frac{{\omega_{\mathrm{a}} \cos \left( {\pi - \alpha } \right)}}{{\omega_{\mathrm{a}} }} = - f_{3} \left( {\gamma ,\lambda } \right) \Rightarrow \alpha = \pi - \arccos \left[ { - f_{3} \left( {\gamma ,\lambda } \right)} \right] $$

For a labyrinth similar to Fig. 4, this leads to an angle between the ducts of 73°. An analogous computer simulation of a three-duct system revealed exactly the same angle. This once more shows that a two-duct approximation of a three-duct system often gives surprisingly accurate results.

*(5) Glass models*

In order to discover interesting features of possible flow in the SCD system that are difficult to study in a mathematical way, we have studied the flow in glass models of an odd shape in which the shape of ducts is exaggerated or otherwise changed. This is allowed under certain conditions of Reynolds numbers and observability of flows that are not explained here (cf. Muller and Verhagen (2002c)). The flow is visualized using polystyrene spheres within the fluid that the glass ducts contain.

**Fig. 8:**
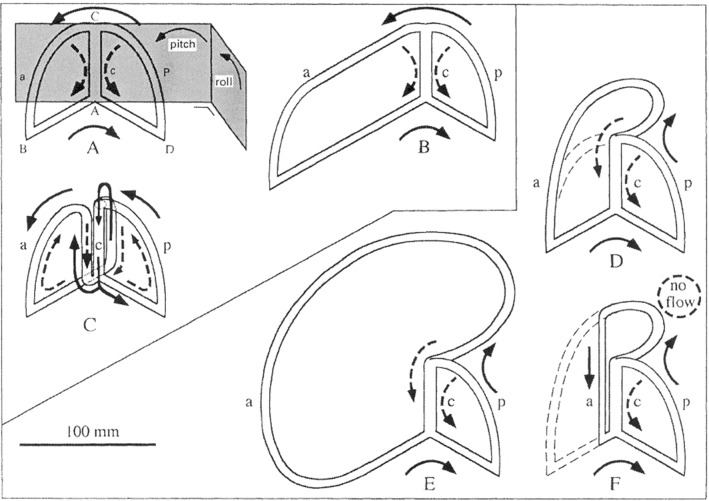
Diagrammatic survey of fluid flow in glass models of two-duct semicircular ducts. The flow in pitch rotation is shown by continuous arrows, the flow in roll by broken arrows. Abbreviations are the same as in Fig. 1. **A**: original model with equal ducts a and p. Reference planes with rotations are shown. **B**: Enlargement of duct a, as in the angler fish (*Lophius*). **C**: the same system as in A but points A and C reversed. **D**: duct a enlarged, as in birds. **E**: the same system as in D but duct a exaggeratedly enlarged. **F**: the same system as in D but part of duct a has been cut off. In all cases, except F in roll, the flow in ducts a and p is the same: after Muller and Verhagen (2002c)

Figure 8A shows the two-duct SCD system with reference planes, as studied above. In Fig. 8B and D, E, one duct is greatly enlarged, as, for example, present in the angler fish (B, *Lophius*) and in birds (8D, and much exaggerated: 8E). In Fig. 8C, the points of confluence of the duct with the crus commune have been interchanged. In Fig. 8F, a bird-like labyrinth is drawn in which the anterior part is lacking.

It is striking that, apart from diagram (8F), the flow in the *ducts* is conserved, even in Fig. 8C.

This flow conservation implies that size and shape may vary to a large extent but the actual shape of the SCD system is apparently determined by external factors, such as the shape of skull and brain.

*(6) Optimal two*-*duct labyrinth shapes.*

**Fig. 9:**
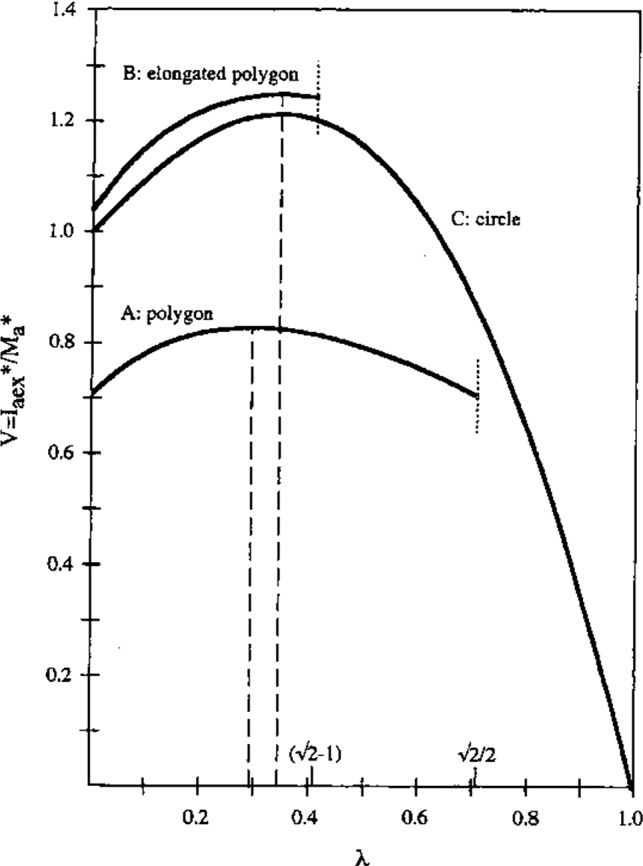
Normalized external impulse ${\mathbf{I}}_{\mathrm{aex}}$ as a function of $\lambda$ for various two-duct SCD systems of different geometries. For a rather small region of $\lambda$, the external impulses are maximal, which is *only* the case for labyrinth shapes in which the connection between the ducts a and p shows a dip. For smooth connections, no optimal value of $\lambda$ exists

Determination of the external impulses (in a normalized form) for a variety of shapes of a two-duct SCD system (e.g. two combined polygonal shapes, or the combination of two circular ducts and a common part) may lead to an optimal value for *λ* (Fig. 9). This is always achieved for a labyrinth shape with a dip between the two ducts, as shown in Fig. 1AA. When a dip is lacking (Fig.1), no optimum for λ can be found. This closely corresponds to the fact that in natural systems nearly always a dip occurs. In turn, this suggests that the shape of these natural systems has been more or less optimized by evolution.

Figure 9 shows the normalized external impulse in duct a of Fig. 1 as a function of λ. A maximal value of this impulse is obtained for about the same *λ* for various labyrinth shapes. Because of the length of the derivations (Muller and Verhagen 2002b), which includes a lot of necessary but uninteresting algebra, the graph is presented without further comment.

*(7) Optimal positioning of SCD systems in the head.*

The equations (16) allow the evaluation of an optimal positioning of the vertical semicircular ducts in the animals’ head (i.e. the ducts a and p of Fig. 1). To achieve this, the anterior duct is placed under a variable angle µ with the midline. The angle between the anterior and posterior ducts α may be chosen as a fixed value of 90° (which simplifies the analysis) or may be chosen freely in more general considerations.

Then

22 $$ \mu = \frac{\pi - \alpha }{2} $$

for animals that move in the pitch direction. So, the vertical ducts should always be positioned symmetrically and obliquely with respect to the midline, and, to the author’s knowledge, this indeed can be observed in all vertebrates.

*To conclude, in Sects.* *6**and* *7**it has turned out to be possible to derive analytical expressions for a simplified two*-*duct SCD system. From these expressions, important insights could be obtained concerning flow distribution at a variety of rotations and geometries of the SCD system. Additional insights have been obtained concerning optimal shapes of the SCDs and about their optimal positions inside the animal’s head.*

In Sects. 8 and 9, three-dimensional labyrinths are no longer considered. Instead, we focus on single-duct systems.

## The SCD system as a transducer

**Fig. 10:**
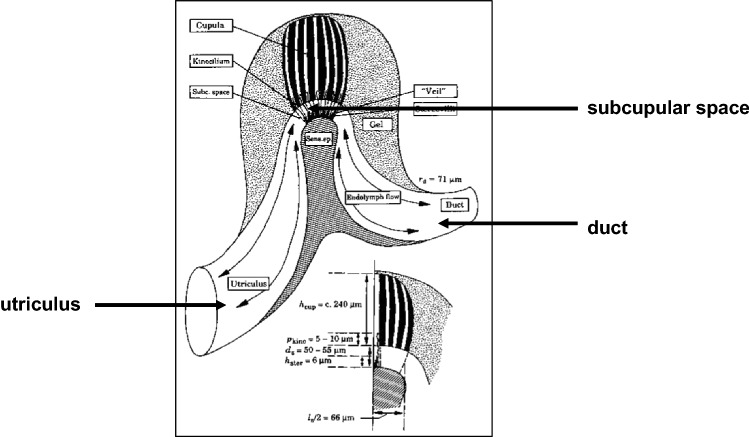
Diagrammatic drawing of the morphology of the mechano-electrical transduction organ inside the ampulla; after Muller (1994). The cupula is, at its distal end, anchored to the roof of the ampulla. It consists of a mucopolysaccharide body in which longitudinally microtubuli are running. It is embedded in a mass of less viscous mucopolysaccharide which forms a continuous border for the endolymph with the walls of utriculus and duct. The crista ampullaris epithelium contains hair cells. Each cell bears a hair bundle which is enclosed in a veil-like tube between the crista and the cupula (i.e. the subcupular space). A hair bundle consists of a single kinocilium inside containing a 9 + 2 structure of dynein filaments, and an array of a variable amount of stereovilli each anchored with the cell body by an actin filament. The kinocilium of a hair bundle partly penetrates in a microtubulus of the cupula. The kinocilium may longitudinally slide inside the microtubulus. There is also enough space for Brownian motion perpendicular to the bundle. Mechanical stimuli may cause the kinocilium to undulate (Rüsch and Thurm 1990)

So far, the main characteristics of a coupled SCD system have been studied. In the present section, some features of the mechano-electrical transduction system in the ampulla will be addressed. It is therefore sufficient to consider a single-duct SCD system. Figure 10 shows a schematic picture of the ampullar system; after Muller (1994). Roughly, the sensory epithelium is covered by a gelatinous cupula. The hair bundles are composed of a single kinocilium of about 70 μm length and an array of stereovilli, the largest are about 20 μm long. The kinocilia penetrate into small tubules of the cupula over a length of about 10 μm. They are able to slide within these tubuli. The kinocilia may undulate under the influence of mechanical stimulation.

Between the sensory epithelium and the cupula, a narrow *subcupular space* exists in which endolymph flow is possible. At its other side, the cupula is anchored into the roof of the ampulla. Much debate has occurred about the function of the cupula. Traditionally, the cupula has been considered as a swinging or sliding structure that mediates the endolymph flow in the ducts into a stimulus of the hair bundles (cf. Muller 1994). In the seventies, Dohlman (e.g. 1980) has argued that the hair bundles were *directly* stimulated by the endolymph flow that was forced through the subcupular space. The cupula would then act as a protecting structure against overload. This theory has been confirmed by experiments of Suzuki et al. (1984). Unfortunately, the above discoveries have been largely neglected in current literature. Since the author’s findings have supported Dohlman’s subcupular space theory as well, it is adopted here.

*(1) Optimization of sensitivity.*

It is well known that the speed of a water jet flowing from a tube can be enlarged by narrowing the tube opening. Obviously, further narrowing of this opening finally stops the flow. Muller (1994) has shown that a comparable phenomenon applies to the flow in the subcupular space. This implies that the length and width of the subcupular space may be attuned to an optimal value. To investigate this optimality, a model has been made of a single duct circuit composed of a duct part d, a utricular part u and a subcupular space part s with different dimensions. Analogous to Eq. (5), the matrix for the initial velocities is

23 $$ \left[ {\begin{array}{*{20}l} {{{m_{d} } \mathord{\left/ {\vphantom {{m_{d} } {A_{d} }}} \right. } {A_{d} }}} \hfill &\quad {{{m_{s} } \mathord{\left/ {\vphantom {{m_{s} } {A_{s} }}} \right. } {A_{s} }}} \hfill &\quad {{{ - m_{u} } \mathord{\left/ {\vphantom {{ - m_{u} } {A_{u} }}} \right. } {A_{u} }}} \hfill \\ { - A_{d} } \hfill &\quad { - A_{s} } \hfill &\quad 0 \hfill \\ 0 \hfill &\quad {A_{s} } \hfill &\quad {A_{u} } \hfill \\ \end{array} } \right]\left[ \begin{aligned} {\dot{\mathbf{x}}}_{{\mathbf{d}}} \left( {\mathbf{0}} \right) \hfill \\ {\dot{\mathbf{x}}}_{{\mathbf{s}}} \left( {\mathbf{0}} \right) \hfill \\ {\dot{\mathbf{x}}}_{{\mathbf{u}}} \left( {\mathbf{0}} \right) \hfill \\ \end{aligned} \right] = \left[ \begin{aligned} \left( {{{{\mathbf{I}}_{\mathrm{dex}} } \mathord{\left/ {\vphantom {{{\mathbf{I}}_{\mathrm{dex}} } {A_{d} + {{{\mathbf{I}}_{\mathrm{sex}} } \mathord{\left/ {\vphantom {{{\mathbf{I}}_{\mathrm{sex}} } {A_{s} - {{{\mathbf{I}}_{\mathrm{uex}} } \mathord{\left/ {\vphantom {{{\mathbf{I}}_{\mathrm{uex}} } {A_{u} }}} \right. } {A_{u} }}}}} \right. } {A_{s} - {{{\mathbf{I}}_{\mathrm{uex}} } \mathord{\left/ {\vphantom {{{\mathbf{I}}_{\mathrm{uex}} } {A_{u} }}} \right. } {A_{u} }}}}}}} \right. } {A_{d} + {{{\mathbf{I}}_{\mathrm{sex}} } \mathord{\left/ {\vphantom {{{\mathbf{I}}_{\mathrm{sex}} } {A_{s} - {{{\mathbf{I}}_{\mathrm{uex}} } \mathord{\left/ {\vphantom {{{\mathbf{I}}_{\mathrm{uex}} } {A_{u} }}} \right. } {A_{u} }}}}} \right. } {A_{s} - {{{\mathbf{I}}_{\mathrm{uex}} } \mathord{\left/ {\vphantom {{{\mathbf{I}}_{\mathrm{uex}} } {A_{u} }}} \right. } {A_{u} }}}}}}} \right) \hfill \\ 0 \hfill \\ 0 \hfill \\ \end{aligned} \right] $$

To simplify (23), we omit the utricular part. As shown by Muller (1994), this condition can be relaxed subsequently. What remains yet is a duct circuit composed of a single semicircular duct and a subcupular space (duct) in the ampulla. Then, the initial velocity in the subcupular space can be derived (Muller 1994) from (23),

24a $$ {\dot{\mathbf{x}}}_{s} \left( 0 \right) = \left( {{\varvec{\upomega}} \times {\mathbf{r}}} \right)\frac{{1 + \frac{{l_{\mathrm{d}} }}{{l_{\mathrm{s}} }}}}{{1 + \frac{{l_{\mathrm{d}} }}{{l_{\mathrm{s}} }} \cdot \frac{{A_{\mathrm{s}} }}{{A_{\mathrm{d}} }}}} $$

To focus on only relevant quantities, Eq. (24a) is normalized to (indicated by a star)

24b $$ {\dot{\mathbf{x}}}_{s} \left( 0 \right)^{ * } = {{{\dot{\mathbf{x}}}_{s} \left( 0 \right)} \mathord{\left/ {\vphantom {{{\dot{\mathbf{x}}}_{s} \left( 0 \right)} {\left\| {{\varvec{\upomega}} \times {\mathbf{r}}} \right\|}}} \right. } {\left\| {{\varvec{\upomega}} \times {\mathbf{r}}} \right\|}} $$

Similarly, the EoM may be solved to obtain the fast time constant (*T*_2_) and the maximal endolymph displacement $x_{s,\mathrm{max} }$ (see Sect. 5). Yet, these quantities are normalized too.

24c $$ T_{2}^{ * } = {{\left( {{M \mathord{\left/ {\vphantom {M F}} \right. } F}} \right)} \mathord{\left/ {\vphantom {{\left( {{M \mathord{\left/ {\vphantom {M F}} \right. } F}} \right)} {\left( {{{A_{\mathrm{d}} \rho } \mathord{\left/ {\vphantom {{A_{\mathrm{d}} \rho } {8\pi \eta }}} \right. } {8\pi \eta }}} \right)}}} \right. } {\left( {{{A_{\mathrm{d}} \rho } \mathord{\left/ {\vphantom {{A_{\mathrm{d}} \rho } {8\pi \eta }}} \right. } {8\pi \eta }}} \right)}} = 8\pi \eta {{T_{2} } \mathord{\left/ {\vphantom {{T_{2} } {A_{\mathrm{d}} }}} \right. } {A_{\mathrm{d}} }} $$

24d $$ x_{s,\mathrm{max} }^{ * } = \left\| {\dot{x}_{s} \left( 0 \right)^{ * } } \right\|T_{2}^{ * } = \left( {{{8\pi \eta } \mathord{\left/ {\vphantom {{8\pi \eta } {A_{d} \left\| {\omega \times r} \right\|}}} \right. } {A_{d} \left\| {\omega \times r} \right\|}}} \right)x_{s,\mathrm{max} } $$

**Fig. 11:**
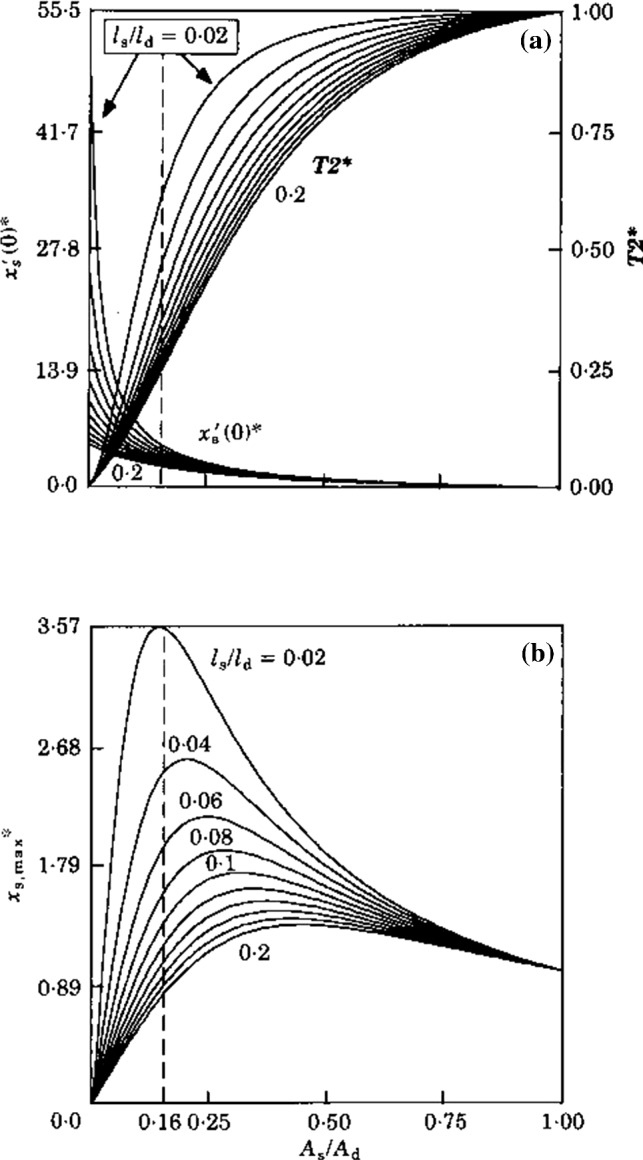
**a** Initial endolymph velocity $\dot{\varvec{x}}(0)$, fast time constant *T*_2_ and (**b** maximal endolymph excursion $\varvec{x}_{\mathrm{max} } = \dot{\varvec{x}}(0) \cdot \varvec{T}_{2}$. All quantities on the ordinates are normalized. On the abscissa, the ratio of the cross-sectional areas $\frac{{A_{\mathrm{s}} }}{{A_{\mathrm{d}} }}$ of subcupular space (s) and duct (d) is shown. Each line represents values for length ratios of (s) and (d) $\frac{{l_{\mathrm{s}} }}{{l_{\mathrm{d}} }}$. The maxima in diagram (**b**) indicate optimal values of $\frac{{A_{\mathrm{s}} }}{{A_{\mathrm{d}} }}$. The broken line indicates the theoretical optimum that is appropriate to a SCD system. For further explanation, see the main text; particularly, Eq. (24)

A graphical representation of Eqs. (24c) and (24d) is given in Fig. 11A and of Eq. (24b) in Fig. 11B.

Measurements of Ramprashad et al. (1984) confirm the optimal values of length- and cross-sectional area ratios found by our subcupular space model, as indicated by the broken line in Fig. 11.

The movability of the kinocilia and their distal embedding in the cupula have led the author to a hypothesis which he has not published until now. Possibly, the width of the subcupular space might be controlled by rising and falling movements of the cupula caused by combined undulating movements of all kinocilia. This would imply a sensitivity control so as to, for example, protect the delicate mechano-electrical transduction system. Obviously, this is a pure speculation, which is open to future verification.

*(2) Why using Laplace transform* *and related techniques?*

In Sects. 5 and 6, integral transform techniques have been used to solve differential equations that are the equations of motion. In addition to frequency characteristics and other electrical engineering wisdom, another important reason to use Laplace and Fourier transforms exists in that one might be interested in both the time-dependent and the frequency-dependent behaviour of the SCD system. These issues will be addressed in the following subsections.

*(3) Contribution of the different parts of the EoM*

In early studies, the SCD system has been considered as an angular *acceleration* *transducer* (demonstrated by many links in Google Scholar). Later (in the 50s), the application of control theory has led to the view that it acts as an angular *velocity* *transducer* (Mayne 1950). These theories considered the SCD system as a rather separate unit, like an independent module in the plug-in system of an electronic apparatus. Obviously, the above different theories favour the importance of the terms in Eq. (6a). The above features have been discussed in detail by Muller (1990).

Basically, Eq. (6) describes the mechanical behaviour of a damped mass–spring system. In control theory, it is convenient to study its behaviour under sinusoidal stimulation, i.e. to construct frequency characteristics (gain against frequency). Equation (6) is then written in the following time- and Laplace-transformed standard forms [cf. Distefano et al. (1967) and Thomson (1981)]

25a $$ \frac{{{\mathrm{d}}^{2} y}}{{{\mathrm{d}}t^{2} }} + 2\varsigma \omega_{n} \frac{{{\mathrm{d}}y}}{{{\mathrm{d}}t}} + \omega_{n}^{2} y = \omega_{n}^{2} x, $$

and its Laplace transform

25b $$ H\left( s \right) = \frac{Y\left( s \right)}{X\left( s \right)} = \frac{{\omega_{\mathrm{n}}^{2} }}{{s^{2} + 2\varsigma \omega_{\mathrm{n}} s + \omega_{\mathrm{n}}^{2} }} $$

where *ζ* is the damping ratio (a measure for the fading out of the system) and $\omega_{\mathrm{n}}$ is the natural frequency of the system while *x* is the input signal of the system (i.e. its excitation), *y* is the output signal (i.e. its response). *H(s) *=* Y(s)/X(s)* is the transfer function and s is the Laplace variable.

**Fig. 12:**
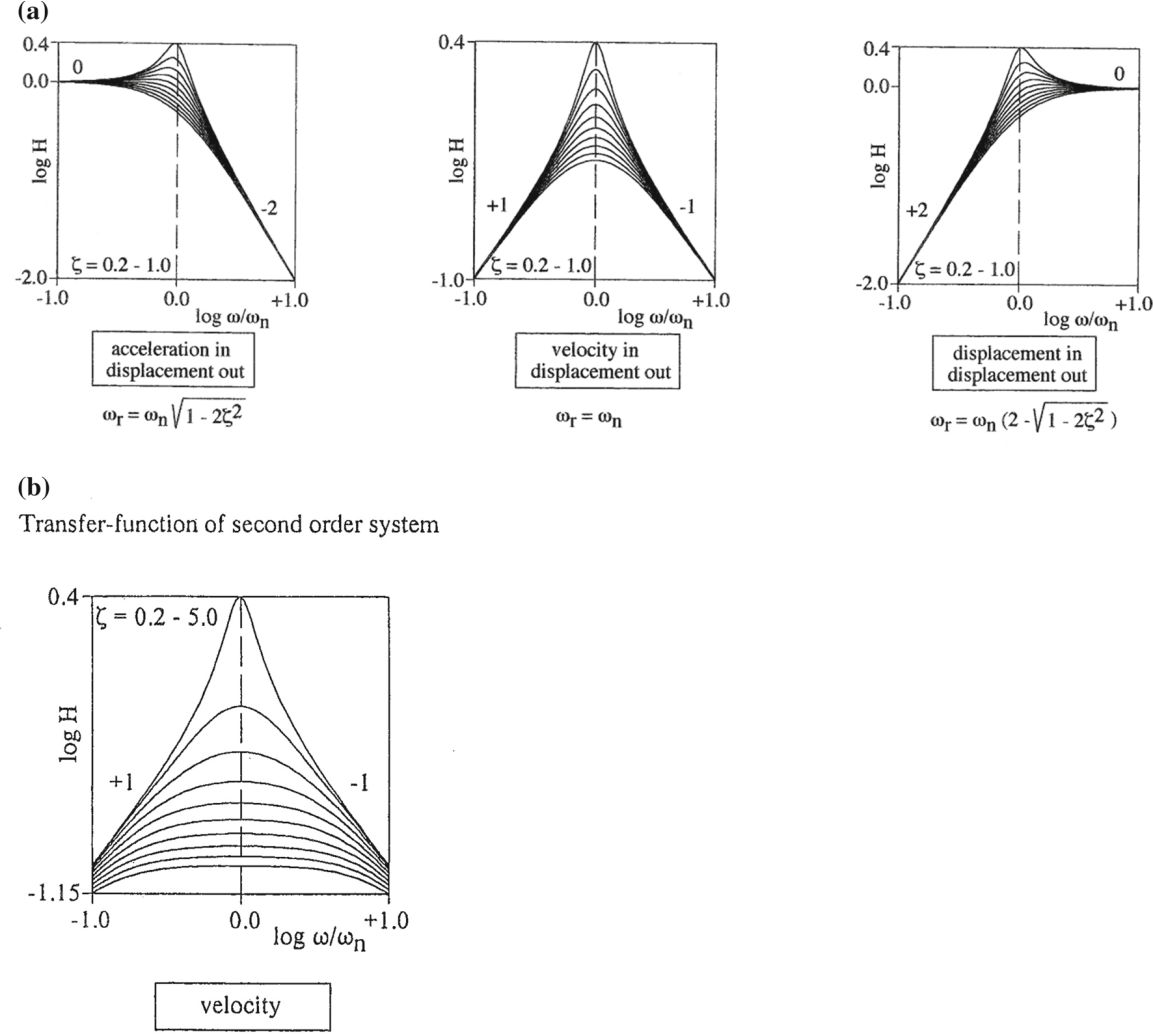
Frequency characteristics of transfer functions (see Eq. 25b) of a second-order system. Normalized frequency is on the abscissa, normalized amplitude is on the ordinate. All graphs represent *the very same system*. In the different graphs, only the *consideration* of this system is different. In **A**, the system in undamped ($\zeta < 1$). The left diagram represents an *accelerometer*. The middle diagram refers to a *velocity meter*. This diagram is conveniently used in radio-engineering. The right diagram stems from a *seismometer*. Here, $\omega$ denotes an angular frequency, $\omega_{\mathrm{n}}$ is the natural frequency, and $\omega_{\mathrm{r}}$ is the resonance frequency. Only for the velocity meter are natural and resonance frequencies equal. In **B** damped systems are considered. The SCD system is highly overdamped ($\zeta \approx 30$), i.e. much more damped than the lowest curves in **A** (*ζ* = 1.0). For such systems, *time constant separation* occurs, which is already visible in the lowest curve of **B**

Figure 12 shows frequency characteristics (amplitude vs. frequency) of these equations for values of *ζ* = 0.2–5.0. This wide range has been chosen to provide a general view of the behaviour of a mass–spring system, which is also applicable to a variety of biological mechanoreceptors. In Fig. 12A, three different views of *the same mass*–*spring system* are given. The left panel of Fig. 12A considers the output displacement when accelerations are applied as input signal. The middle panel gives an analogous consideration when the input is a velocity signal. The right panel gives a view of the system for displacements as input. It is important to notice that for the three above considerations, the mass–spring system is the very same. This means that *no three different sensors* exist for acceleration, velocity and displacement.

Figure 12B is an extension of the velocity plot of Fig. 12A (middle panel) for larger values of *ζ*. For *ζ* = 5 (lowest graph), separation of corner frequencies (i.e. the inverses of the time constants), can be observed, forming a plateau-like curve. The damping ratio of SCD system is about *ζ* = 30. So, here the time constants differ a factor of about 1000, forming a plateau with a much larger bandwidth. For a human-like labyrinth, the corner frequencies are in the range of *f*_1_= 0.05 Hz and *f*_2_= 30 Hz (Muller 1999). The useful frequency range is 0.5–5.0 Hz [that is, nobody can swing the head at the corner frequencies (Muller 1999]: Fig. 12. These values concur with the time constants mentioned in Sect. 5.

Yang and Hullar (2007) have given a statistical analysis of vestibular nerve afferents in mice. They reported a bandwidth of about *f*_1_= 0.1 Hz and *f*_2_= 10 Hz. Hullar and Minor (1999) reported a bandwidth of 2–20 Hz for the chinchilla.

The above results show that the SCD system cannot be separated into different modules. Instead, it is a single sensor that *simultaneously measures displacement, velocity and acceleration*. It does not matter at which neuronal level integrations should occur. Contrary to the widely accepted classical view that the SCD system acts as a velocity transducer that senses sinusoidal head rotations (e.g. Hullar and Minor 1999; Mallery et al. 2010), strong arguments exist that its major function is *not* to measure sinusoidal movements. Instead, measurements of *step*-*like* movements are made [discussed in more detail by Muller (1990)], as can be underpinned by observation of head movements of a great many different vertebrate species, including man. In this sense, the SCD system fulfils two functions. As already noted previously, at a timescale of *T*_2_, it is a “measuring device”. At a timescale of *T*_1_, it is a “mechanical memory”. The widely used term *velocity* *storage* is more applicable to the neuronal processing of the mechanical output signal of the SCD system. This is outside the scope of the present paper.

*To conclude, the analysis in Sect.* *8.1 strongly supports the subcupular space theory. In short, the endolymph flow presses directly to the cilia of the hair cells in the subcupular space. In turn, the cupula is moved by the cilia. This is contrary to the classical and still widely adopted view that the flow moves the cupula and the cilia of the hair cells are moved by the cupula.*

*In Sects.* *8.2 and 8.3, it has been argued that the SCD system does not act as a transducer sensing the velocities of sinusoidal movements. Instead, evidence has been presented that it can be considered as a transducer that senses, in a rather complex way, simultaneously accelerations, velocities and displacements resulting from sudden, step*-*like, head movements. The above conclusion is applicable only to the hydrodynamic behaviour of the system of semicircular ducts. Neuronal processing leading to body movements is not considered as it is beyond the scope of this paper.*

## Brownian motion and hair cell design and sensitivity

Brownian motion is the phenomenon of irregular movements of tiny particles in water (or another fluid) due to bombardments of water-molecule clusters. Robert Brown discovered it in 1827 while studying cell organelles in plants and observing the movements of these organelles under a microscope. Finally, Einstein (1907) physically explained Brownian motion: see below. In the next section, Brownian motion of hair bundles of mechanoreceptor cells will be discussed.

The physical properties of the hair cells of the SCD system are remarkably different from those of the hair cells of other octavo-lateralis hair cell receptors. Generally, the hair bundles of the cristae are about 70 µm long. This is about 10 times the length of bundles of other hair cell receptors. The peak excursions of the hair bundles of SCD cells are in an order of magnitude of several micrometres (Rabbitt et al. 1995). These excursions are about 500 times larger than the nanometre excursions of other bundles.

The frequencies relevant to SCD receptors range from ca. 0.05 Hz to several tens of Hz, although there is little evidence that the very low frequencies (below a frequency of ca. 0.2 Hz; cf. Figure 12) are biologically relevant. To compare, in whales the frequency domain of auditory and lateral line receptors ranges from ca. 10 Hz to 150 kHz. Thus, two rather distinct groups of hair cells exist: slow and insensitive ones and fast and sensitive ones. Muller et al. (2016) have shown that this serves to avoid Brownian motion overload in the low-frequency regime. Here, Rüsch and Thurm (1990) have reported a r.m.s. Brownian motion noise amplitude of 70 nm. This is an unusually large value. Such a noise would mask the adequate signals of fast hair cell receptors, if they would operate in this regime. The maximal tip excursions of ampullar hair bundles are about a factor 100 larger than the ones of Brownian motion, so that here the contribution of noise is unimportant.

To achieve and underpin the above results, an ampullar hair bundle has been modelled as a stiff rod that is, at its base, elastically connected to the cell body. For the analysis of Brownian motion, it is convenient to start with a power equation, as derived by Muller et al. (2016) from Reif (1965):

26a $$ \tau \dot{\lambda } + \lambda = 4D\tau \quad [{\mathrm{m}}^{2} ] $$

where

26b, c, d $$ \lambda = x^{2} \quad D = \frac{{k_{\mathrm{B}} T}}{\gamma }\quad \tau = \frac{\gamma }{2\kappa } = \frac{{T_{\mathrm{c}} }}{2} $$

The quantity *x* [m] is the tip excursion of the hair bundle. The quantity *λ* [${\mathrm{m}}^{2}$] is not literally the power but is a measure for it. *D* [${\mathrm{m}}^{2} /{\mathrm{s}}$] is the diffusion constant, $k_{\mathrm{B}}$ is Boltzmann’s constant [J/°K], *T* is the absolute temperature [°K], *γ* [kg/s] is the coefficient of friction for a prolate rod and *κ* [N/m] is the spring constant of the elastic part at the base of the rod. $T_{\mathrm{c}}$ and *τ* [s] are time constants.

The asymptotic solution to Eq. (26) is shown in Eq. (27). This equation represents the Brownian motion’s amplitude spectrum $X^{ * } \left( f \right)$,

27 $$ X^{ * } \left( f \right) = \frac{1}{\pi \sqrt 2 }\sqrt {\frac{{k_{\mathrm{B}} T}}{\kappa }} \cdot \left[ {A\left( {\frac{1}{{\sqrt {\pi^{2} f^{2} a^{2} T_{\mathrm{c}}^{2} + 1} }}} \right) + B\left( {\frac{1}{{\sqrt {\pi^{2} f^{2} b^{2} T_{\mathrm{c}}^{2} + 1} }}} \right)} \right]\quad \left[ {\mathrm{m}} \right] $$

where *A*, *B*, *a* and *b* are constants, chosen in the calculation to approximate a square-root expression by the sum of two exponentials, (*A* + *B* = 1). The details of the above calculations can be found in Muller et al. (2016).

**Fig. 13:**
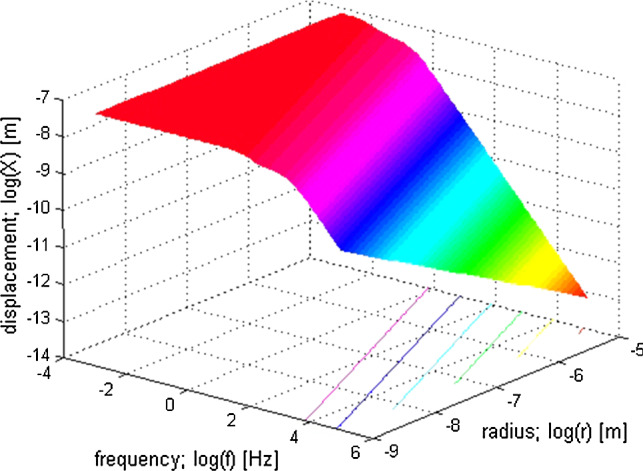
Three-dimensional frequency characteristic of Brownian motion noise of a SCD hair bundle. All axes are logarithmic. The lines at the bottom of the cube are projections of the three-dimensional plot. The r.m.s. values of BM noise (in [*nm*]) are given in the panels. For further explanation, see the main text; particularly, Eq. (27)

Equation (27) looks graphically like a somewhat deformed low-pass filter function: see Fig. 13. It reveals that for *f* = 0 the low-frequency plateau of (27) is at

28 $$ X^{ * } \left( 0 \right) = \frac{1}{\pi \sqrt 2 }\sqrt {\frac{{k_{\mathrm{B}} T}}{\kappa }} \quad \left[ {\mathrm{m}} \right] $$

A chosen value of the low-frequency plateau (e.g. the 70 nm reported by Rüsch and Thurm (1990)) enables the calculation of the elasticity constant *κ* of a hair bundle.

The famous equation of Einstein (1907) for the mean diffusion of a particle

29 $$ \bar{x} = \sqrt {4Dt} \quad \left[ {\mathrm{m}} \right] $$

provides a clue to the value of the low-frequency plateau in Eq. (28). In the frequency domain, formula (29) becomes [derivation analogous to Eq. (27)]

30 $$ X^{ * } \left( f \right) = fX\left( f \right) = \frac{1}{2\pi }\sqrt {\frac{D}{2f}} \quad \left[ {\mathrm{m}} \right] $$

For low frequencies (applicable to an ampullary hair bundle), this formula yields a r.m.s. particle displacement of about 70 nm. This surprisingly well agrees with the choice of 70 nm in Eq. (28) mentioned above.

*To conclude, starting from thermal agitation of water molecules (endolymph),* via *hair bundle size, sensitivity and frequency response, an explanation is given for the existence of the macroscopic guiding structures for endolymph flow that originates from the semicircular ducts. It has been revealed too that two types of hair cells exist,* viz*. slow and insensitive ones vs*. *fast and sensitive ones*

## Size limitations

We will now explore to what extent the physical properties of the SCD system are limiting its size. For this purpose, the simplest approach is to consider a single duct system. It will turn out that considering a system of more ducts is not necessary.

**Fig. 14:**
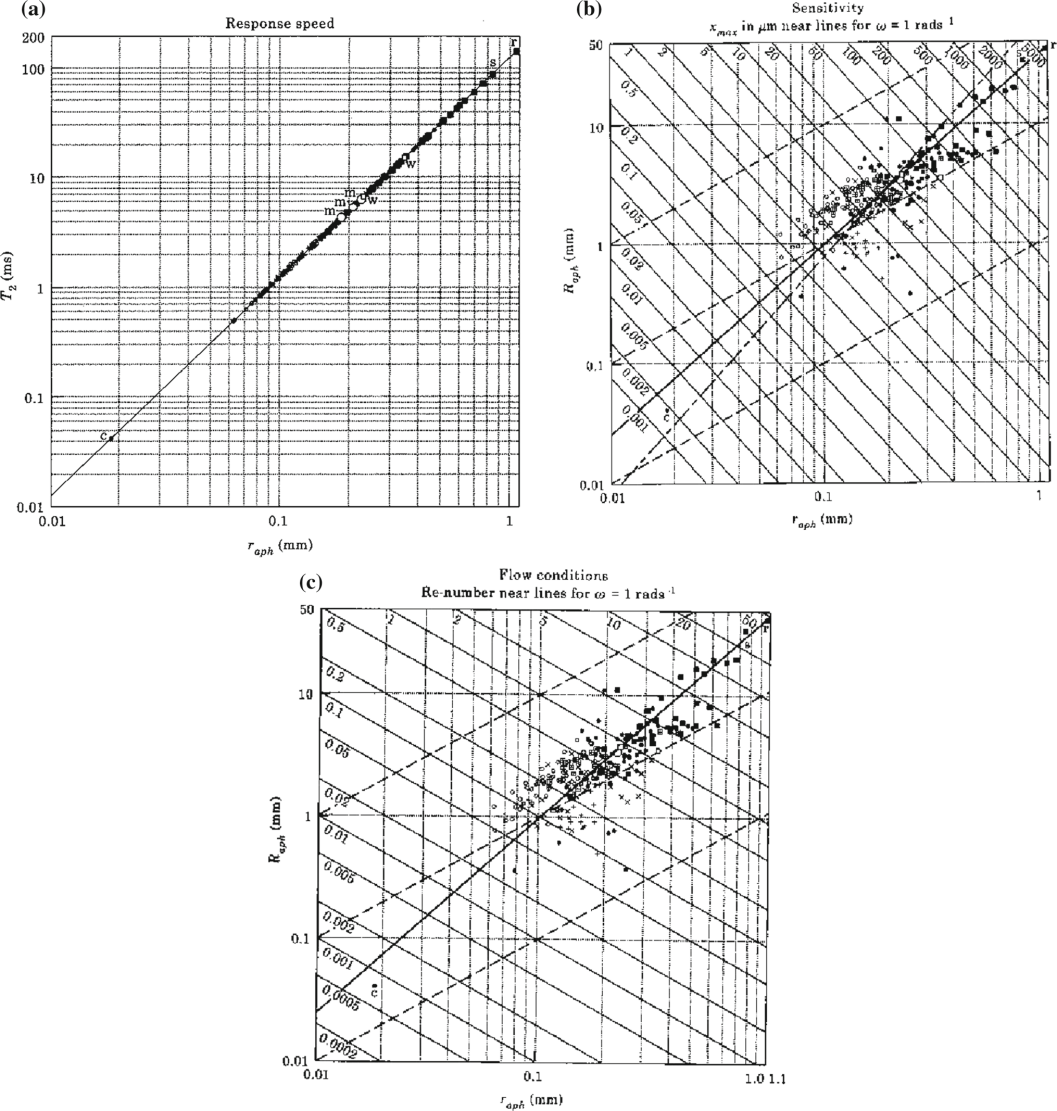
Response speed (**A**), sensitivity (**B**) and conditions for undisturbed endolymph flow (**C**) impose demands and limitations for SCD construction. Characteristics for these quantities are the fast time constant *T*_2_, the maximal endolymph displacement $x_{\mathrm{max} }$ and the angular Reynolds number Re. These quantities are plotted as functions of the circuit radius (*R*) and the radius of the duct cross section (*r*). Note logarithmic axes. In the plots, measurements of (*R*) and (*r*) are given. The experimentally measured radii are plotted for the mean of the three semicircular ducts. For further explanation, see the main text; particularly Eq. (31)

The largest labyrinth is found in the whale shark (*Rhincodon*). This is a very large shark, which can reach a length of about 18 m. The size of its SCD system, containing a large mass of endolymph, makes its response speed very low (*T*_2_ is about 150 [ms], cf. Figure 14A). This would not impose a big problem in whales and giant sharks as their turning speeds are also very low (compare them with a supertanker). SCD size is probably limited too by secondary flow in the plane of the cross-sectional area of the ducts, i.e. perpendicularly to the main flow (Muller 1999).

The labyrinths of whales are generally considered to be very small but this applies to their *relative* size (because whales are very large). Their *absolute* size is comparable to labyrinths of other mammals, including humans (e.g. Kandel and Hullar 2010; Spoor et al. 2002; Muller 1999). Figure 14 presents an overview of measurements of SCD parameters of a variety of vertebrates in which the above values can be verified.

The smallest labyrinth can be found in larvae of fish and some amphibians. These SCD systems contain an extremely small quantity of endolymph fluid (nanolitres). Hence, their sensitivity will also be extremely small (Fig. 14B). Brownian motion noise (Sect. 9) is probably another limiting factor (Muller 1999).

*(1) The basis of limitations*

The main functional quantities of a rotation receptor formed by a circular single-duct circuit are response speed, sensitivity and undisturbed Poiseuille flow. Two morphological parameters are characteristic for such a circuit, viz. the circuit radius *R* and the radius of the canal cross section *r*.

The fast time constant (*T*2) can be considered as a measure of the response speed, whereas the maximum endolymph displacement ($x_{\mathrm{max} }$) is a measure of the sensitivity, see Sect. 5. The (angular) Reynolds number (Re) indicates the extent of laminar Poiseuille flow: for details regarding the angular Reynolds number one may consult, for instance Childs (2011). For Re large enough, the rotational flow becomes turbulent (Childs 2011; Muller 1999). This leads to the following equations (Fig. 14).

31a $$ T_{2}^{{}} = \frac{{r^{2} }}{8\nu },\quad {\mathrm{see}}\;{\mathrm{Eq}}.\;\left( 7 \right) $$

31b $$ x_{\mathrm{max} } = \left\| {{\dot{\mathbf{x}}}(0)} \right\|T_{2} = \left\| {{\varvec{\upomega}} \times {\mathbf{R}}} \right\|\frac{{r^{2} }}{8\nu }\, \Rightarrow \,R = \frac{8\nu }{\omega } \cdot \frac{{x_{\mathrm{max} } }}{{r^{2} }},\quad {\mathrm{see}}\;{\mathrm{Eq}} .\;\left( 8 \right) $$

31c $$ \mathrm{Re} = \frac{{\left\| {{\dot{\mathbf{x}}}(0)} \right\|r}}{\nu } = \frac{{\left\| {{\varvec{\upomega}} \times {\mathbf{R}}} \right\|r}}{\nu }\, \Rightarrow \,R = \frac{\nu }{\omega }\frac{\mathrm{Re}}{r}, $$

from the definition of Re; see above.

Figure 14 shows these relationships as functions of r and R with isolines for $x_{\mathrm{max} }$ and Re. In these graphs, size measurements of a variety of SCD systems have been plotted demonstrating the approximate size range existing in vertebrates (Muller 1999).

Generally, fishes, and particularly sharks, have very large labyrinths. These observations correspond closely to the theoretical limits imposed by Eq. (31) as has been explained in the above introductory paragraphs of Sect. 10. An extensive discussion can be found in Muller (1999).

*(2) Allometric relationships*

Why do fishes have considerably larger labyrinths than other vertebrates? To find an answer for this question, the study of allometric relationships may be helpful. Measurements of SCD sizes and measurements of masses of the corresponding animals lead to the following empirical expressions (Muller 1999):

32a $$ {\mathrm{For}}\,{\mathrm{mammals}}:\quad R = 1.7644\,{\mathrm{m}}^{0.1157} $$

32b $$ {\mathrm{For \,fishes}}:\quad \quad R = 4.5487\,{\mathrm{m}}^{0.2885} $$

**Fig. 15:**
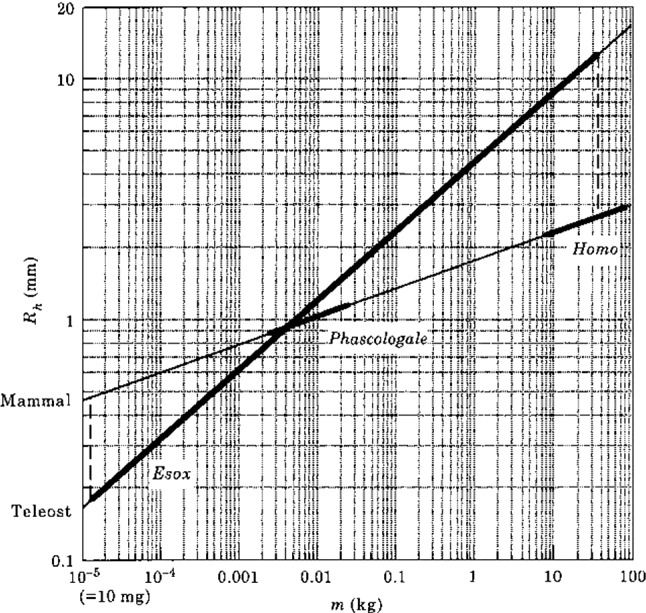
Plot of the circuit radius *R* as a function of the animal’s mass *m*, showing allometry. The three bold regions denote values for pike (*Esox*), a small mammal (*Phascologale*) and man (*Homo*). For further explanation, see the main text; particularly, Eq. (32)

Figure 15 shows these relationships (32) graphically. The bold lines indicate the mass ranges during lifetime of some animals. A pike (*Esox*) grows from an about free swimming larva with a mass of 13 [mg] to an adult weighing 35 [kg] (a popular but perhaps rather exaggerated angler dream), so that it increases by about 7 decades in mass. For mammals, mass increase during active life is about 1 decade. This is a dramatic difference. That is to say, when man would follow the allometric line of a pike, it would have a much larger labyrinth than the pike.

A freely swimming pike larva is a predator. To sense rotations during swimming, it should possess a functional labyrinth. Its circuit radius (*R*) is then about 0.2 [mm]. Mass increase along its allometric line implies that the circuit radius of the adult is about 15 [mm] which is a very large value compared to mammals.

Why are these allometric lines so different? Muller (1999) argued that this is a consequence of brain size and available room inside the skull. Shortly, fishes have small brains. In their neurocranium, there is plenty of room for a labyrinth. One can imagine that in these animals there has been a tendency to evolve large, maximally sensitive labyrinths. Mammals and birds possess large brains, almost completely filling their braincase and therefore leaving limited room for other organs. So, this should impose an evolutionary constraint.

*In the above section, it has been shown that response speed, sensitivity and undisturbed flow are important functional demands to an SCD system. These quantities could be evaluated by plotting data of circuit* *and duct radius against each other giving clues to size limitations, i.e. limitations of the smallest and the largest possible SCD systems. An evolutionary interpretation of the very large difference in allometric relationships between fishes and tetrapods has been proposed.*

## Evolution

We have now already gradually arrived at evolutionary aspects. In this final section, we will focus a bit more on biology and a short overview of SCD evolution will be given, starting from simple hair cells to different designs of SCD systems.

In ascidians (*Urochordata*), at the entrance of the oral siphon, so-called *coronary organs* have been found (Caicci et al. 2007). They contain hair cells that show a remarkable similarity to octavo-lateralis hair cells of vertebrates. They function in sensing water and particle flow. The above authors argued that these cells are the best candidates for the fast and sensitive type hair cells mentioned in Sect. 9. The slow and insensitive cells should then have been evolved from these fast and sensitive ones. The non-vertebrate chordates do not possess labyrinth organs. They possess various types of hair cell mechanoreceptors at different places on their body but the coronary organs resemble neuromasts and lateral line cells of vertebrates most closely. Labyrinth organs are vertebrate structures. In ontogeny, the labyrinth develops from an *otic placode* which invaginates into a vesicle. So, it is from epidermal origin, which demonstrates its relationship with hair cell mechanoreceptors (e.g. *neuromasts*) of the skin. The above evolutionary information is extremely relevant and supportive of the discussion of Brownian motion addressed in Sect. 9.

In *Agnatha*, the hagfish (*Myxine*) possesses a labyrinth of a highly aberrant form (Jørgensen et al. 1998) which is not further discussed here. It has been thought a long time that other *Agnatha* possess two semicircular ducts. Muller (2000) argued that this cannot be the case because Löwenstein and Thornhill (1970) recorded nerve signals as a response to horizontal rotation. Muller (2000) proposed a horizontal flow circuit in the labyrinth of *Agnatha* and argued that a three-duct SCD system is the basic design, and not a two-duct one. This probably has been present already in the earliest known vertebrates (e.g. *Ostracodermi*). In 2014, a horizontal duct was discovered in a lamprey (*Petromyzon*) which has been overlooked for more than a century (Maklad et al. 2014).

In sharks and rays (*Elasmobranchii*), the posterior duct has become rather separate, although a connection of variable size with the rest of the SCD system exists. In some of these animals, a sensor with a separate nerve has been found. The function of this organ is still unknown. The anterior and posterior ducts are hydrodynamically coupled, as described in Sect. 4. The rabbit fish (*Chimaera*) is the only elasmobranch that possesses a SCD system of conventional design (Fig. 1) possibly reflecting the primitive situation.

The SCD system in other vertebrates is composed of three hydrodynamically coupled ducts, as described in Sect. 3, although in some species (i.e. dolphins and whales) the ducts are much reduced in size and some ducts are even closed. Spoor et al. (2002) obtained evidence that this provides protection against overstimulation of the SCD system in these acrobatic and three-dimensionally moving animals.

**Fig. 16:**
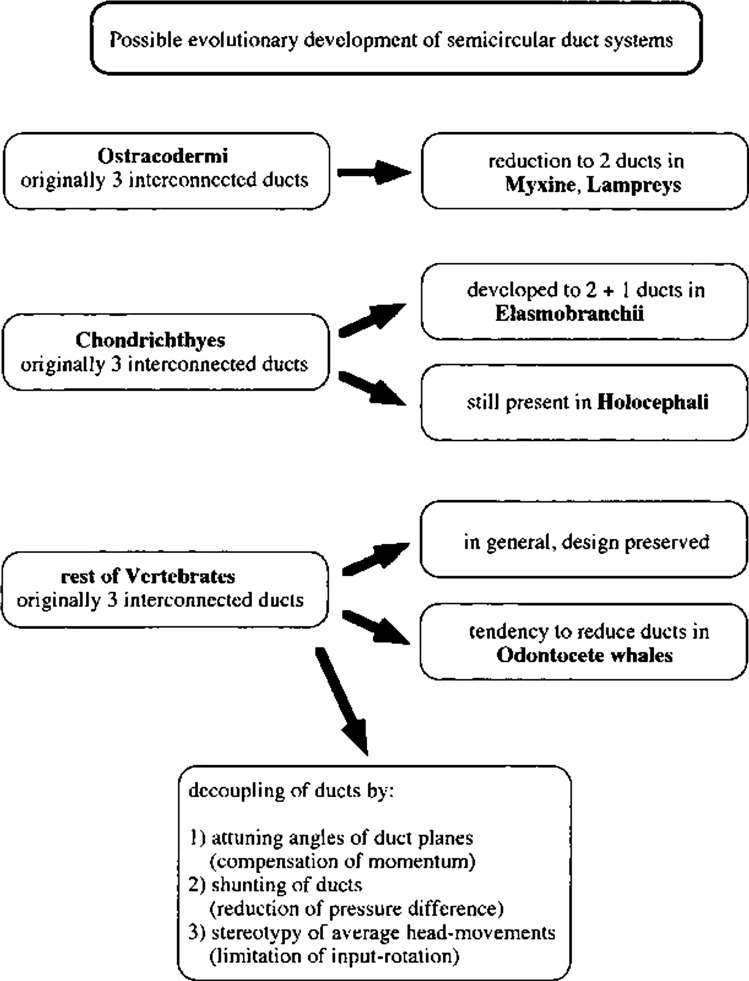
Possible scenarios for labyrinth evolution. Evidence has been found that the original design of the SCD system was composed of all three ducts. Two-duct systems probably evolved from the three-duct one. Elasmobranchs possess a unique construction with a practically separate posterior duct. In whales and dolphins, ducts may be substantially narrowed and are probably not functional. In birds, the anterior duct is greatly enlarged. The functionality of these constructions is still largely unknown. For more explanation, see the main text and Muller (2000)

Figure 16 presents an overview of possible semicircular duct evolution. In the authors view, the basic design is an SCD system composed of three ducts. Other shapes of the SCDs found in different vertebrates are possibly derived from this three-duct system.

## General conclusions

*The present review demonstrates that it is highly fruitful to combine biological features with physical laws and mathematical analysis. Let us focus on a summary of what we have seen.*

*The analysis of a three*-*dimensional SCD system with mutually connected ducts was new at the time of its publication (1988). The analytical approach of a two*-*duct SCD system allowed quantitative determinations of flow distributions at different rotations and about the influence of duct positions and sizes on it. In the evolution* *section, it was argued that a three*-*duct SCD system is the basic design and that other constructions in evolution are derived from this design.*

*In* Sect. *7.6, it was found that an optimal SCD system always shows a dip between the vertical ducts.*

*In* Sect. *7.7, optimal SCD constructions and the positioning of the SCDs in the animal’s head have been evaluated.*

*The results obtained in* Sect. *8.1 strongly support the subcupular space theory in which endolymph flow directly moves the cilia of the hair cells, and in turn the cilia move the cupula.*

*Viewing the analysis of the SCD system as a transducer led us to new insight into its function so that we could reject the concept that it acts as a pure velocity transducer.*

*The study of Brownian motion reveals that two types of hair cells exist,* viz*. slow and insensitive ones and fast and sensitive ones. The first type is found in the ampullae of the SCDs and, to a lesser extent, in the otolith* *organs, whereas the second type is found in the auditory organ and the lateral line. In the evolution* *section, it has been argued that the insensitive type has evolved from the sensitive one.*

*Finally, allometric relationships and size limitations have been considered based on measurements of the SCDs together with their underlying physical requirements. The conspicuous size difference between fish* *and tetrapod SCD systems as has been hypothesized, i.e. this possibly was determined in evolution by brain* *size in relation to the available room in the skull.*
